# Validation of the GlutenTox^®^ ELISA Rapid G12 Test Kit for Determination of Gluten in Select Non-Heat-Processed Matrixes and Heat-Processed Matrixes: AOAC *Performance Tested Method*^SM^ 042301

**DOI:** 10.1093/jaoacint/qsad081

**Published:** 2023-07-17

**Authors:** Carlos Galera, Claudia Salagre, Ana López

**Affiliations:** Quality Department, Hygiena Diagnóstica España, S.L.U, Calle Cañada Real 31-35, P.I. Parque Plata, Camas (Sevilla), 41900, España; Quality Department, Hygiena Diagnóstica España, S.L.U, Calle Cañada Real 31-35, P.I. Parque Plata, Camas (Sevilla), 41900, España; Quality Department, Hygiena Diagnóstica España, S.L.U, Calle Cañada Real 31-35, P.I. Parque Plata, Camas (Sevilla), 41900, España

## Abstract

**Background:**

The GlutenTox^®^ ELISA Rapid G12 test kit is a quantitative method designed for the determination of the immunotoxic fraction of gluten in food samples.

**Objective:**

To obtain AOAC *Performance-Tested Methods*^SM^ certification for the method for the detection and quantification of gluten from wheat, barley, and rye flours in select foods (non-heat-processed) and incurred (heat-processed) matrixes.

**Methods:**

The method was evaluated following the *Guidelines for Validation of Quantitative Gluten Methods, with Specific Examples for ELISA Assays*. The validation study was conducted at Hygiena Diagnóstica España using five food matrixes (soy flour, corn bread, seasoning mix, rolled oats, and evaporated milk) artificially contaminated with gluten from wheat, barley, or rye flour at different concentrations: 0, 5, 10, and 20 mg/kg. For each matrix and gluten contamination level, five or six individually extracted test portions were analyzed. A second bread matrix was prepared by baking a gluten-free bread mix spiked at 0, 20, and 30 mg/kg gluten from wheat, barley, or rye flour for incurred matrix testing. Ten individually extracted test portions were tested for each incurred bread and contamination level of gluten.

**Results:**

The method met the AOAC performance requirements for detection and quantification of wheat gluten in the selected food matrixes, incurred bread sample, and spike levels of wheat gluten, showing an acceptable recovery. When tested with barley and rye flours, most of the results showed acceptable recoveries or a slight overestimation, depending on the matrix and gluten concentration. Method developer and independent laboratory results were comparable.

**Conclusions:**

The validation study demonstrated that the test kit is a reliable, accurate, quick, and easy-to-use method for the detection and quantification of gluten concentration in food and incurred matrixes from wheat, barley, and rye flours.

**Highlights:**

Most reagents provided in the kit are at ready-to-use concentrations.

## General Information

Gluten is a protein fraction from wheat, rye, barley, oats, or their crossbred varieties and derivatives thereof, to which some persons are intolerant and which is insoluble in water and 0.5 M NaCl (1).

An inappropriate response of the immune system to gluten causes celiac disease, a disorder that damages the small intestine, causing the atrophy of the intestinal villi, which interferes with the absorption of nutrients such as proteins, lipids, carbohydrates, mineral salts, and vitamins. This disease leads to diarrhea, vitamin and mineral deficiencies, anemia, and thin bones (osteoporosis) and affects people of all ages.

Currently, the only treatment for celiac disease sufferers is a strict, lifelong gluten-free diet, which presents great difficulties because gluten, in addition to being present in many foods, may also be found in food additives and preservatives.

## Principle

The GlutenTox^®^ ELISA Rapid G12 method is a sandwich ELISA assay that can be used to detect and quantify gluten in food samples.

To solubilize the gluten present in the sample’s matrix, the extraction solution (UGES) (2) provided in the kit is added to the food sample.

After the extraction, the sample’s extract is added to a multi-well plate coated with a monoclonal anti-gliadin antibody (G12) that specifically recognizes the most immunogenic fraction of gluten. After the washing steps, the addition of a second monoclonal anti-gliadin antibody conjugated to HRP (A1-HRP) and the substrate solution (TMB) will allow for the measurement of the signal (color change). GlutenTox ELISA Rapid G12 is a direct method. The higher the concentration of gluten present in the sample, the more intense the signal will be.

### Scope of Method

- *Analyte(s).*—Gluten from wheat, barley, and rye flour.
- *Matrixes.*—Soy flour, corn bread, seasoning mix, rolled oats, evaporated milk, and gluten-free baked bread.
- *Summary of validated performance claims*.—The GlutenTox ELISA Rapid G12 test kit is designed to detect and quantify gluten in processed and non-processed foods listed above at a range of 0.6–200 mg/kg gluten. This range of quantitation is suitable for proposed gluten-free monitoring in the United States and is compliant with current EU regulations and Codex Alimentarius definitions.

### Definitions

- *Repeatability.*—Precision under repeatability conditions. (ISO 5725-2) (Repeatability conditions: Conditions where independent test results are obtained with the same method on equivalent test items in the same laboratory by the same operator using the same equipment within short intervals of time (3).
- *Reproducibility.*—Precision under reproducibility conditions. (ISO 5725-2) (Reproducibility conditions: Conditions where independent test results are obtained with the same method on equivalent test items in different laboratories with different operators using separate instruments (3).
- *Intermediate precision.*—Precision under intermediate conditions. (ISO 3534-2). (Intermediate precision conditions: Conditions where test results or measurement results are obtained with the same method, on identical test/measurement items in the same test or measurement facility, under different operating conditions (4).
- *Linearity*.—Linearity of the method extending beyond the set of standards or calibrators supplied with the kit. Direct and unambiguous relationship between measurement response and concentration.
- *Calibrant*.—A material used for calibration of a measurement procedure.
- *Selectivity*.—Ability of the method to detect analyte without interference from matrix or other components of similar behavior.
- *Relative recovery*.—Recovery is the ratio of the mean candidate method result to the true value or reference method value, expressed as a percentage, [mean_cand_/known spike] × 100 or [mean_cand_/mean_ref_] × 100.
- *Bias*.—Bias is the difference between the candidate method mean result and the true value or reference method value, mean_cand—_known spike or mean_cand—_mean_ref_.
  Bias is the total systematic error as contrasted to random error. There may be one or more systematic error components contributing to the bias.
- *Standard deviation of repeatability*.—s_r_ = $\sqrt{\frac{\sum_{i=1}^{n}{\left(X_{i}-\bar{X}\right)}^{2}}{n-1}}$
- *Relative standard deviation of repeatability*.—RSD_r_ = [s_r_/mean_cand_] × 100
- *LOD*.—Mean + 3.3s_r_, or $\frac{{\bar{X}}_{0}+3.3(s_{b})}{1-1.65m}$
  The lowest concentration or mass of analyte in a test sample that can be distinguished from a true blank sample at a specified probability level, where ${\bar{X}}_{0}$ is the mean concentration value of the blank replicates, *m* is the slope, and *s_b_* is the intercept (3).
- *LOQ*.—Mean + 10s_r_, or 3 × LOD (if linear regression model is used).
  The lowest level of analyte in a test sample that can be reasonably quantified at a specified level of precision (3).

## Materials and Methods

### Test Kit Information

- *Kit name*.—GlutenTox ELISA Rapid G12.
- *Cat. no*.—KIT3075.
- *Ordering information*.—https://www.hygiena.com/.

### Test Kit Components

- GlutenTox ELISA Rapid G12
  1. *Multi-well G12-coated strips*.—12 strips (dividable, 8 wells each).
  2. *Wash solution (10*×*)*.—40 mL bottle.
  3. *Dilution solution*.—120 mL bottle.
  4. *Extraction solution*.—200 mL bottle.
  5. *GlutenTox A1-HRP conjugated antibody*.—15 mL bottle.
  6. *Substrate solution*.—12 mL bottle.
  7. *Stop solution, H_2_SO_4_ 0.5M*.—12 mL bottle.
  8. *5 GlutenTox standards*.—1.25 mL vial each (1.56 to 50 ng/mL gliadin).
  9. *Negative control*.—1.25 mL vial.
  10. *Positive control*.—1.25 mL vial.
  11. *Internal control*.—1.25 mL vial.

### Additional Supplies and Reagents

- *Capped centrifuge test tubes*.—>10 mL.
- *Test vials*.—1.5–2 mL.
- *Disposable gloves*.
- *Distilled water.*
- *Polyphenol pack (KIT3008).*

### Apparatus

- *Analytical scale*.—Accurate to 0.1 g.
- *Tube rotator*.—Or equivalent.
- *Centrifuge*.—Capable of maintaining 2500 × *g* for 10 min.
- *Timer*.
- *Vortex mixer.*
- *Thermostatically controlled water bath*.—Capable of maintaining 50°C.
- *ELISA plate reader*.—Equipped with a 450 nm filter.
- *Mono-channel micropipet.*
- *Multi-channel micropipet*.—Recommended.
- *Micropipet tips*.—Aerosol resistant.
- *Automatic microplate washer*.—Recommended.

### Safety Precautions

The GlutenTox ELISA Rapid G12 contains material that may cause skin or eye irritation. Wear safety glasses with side shields that have been approved under the appropriate government standards. If reagents come in contact with eyes, remove contacts and flush with water for 15 min. Wear gloves when handling materials to avoid contact with skin. Flush skin with plenty of water if accidental contact occurs. Certain chemicals in the kit should not be released into the environment.

### General Preparation

- Carefully read the user manual before performing the assay.
- It is recommended that the instructions described in the manual be followed exactly as described.
- This kit is designed for professional use only.
- Do not mix components from various kits or use reagents or solutions other than those supplied.
- Allow all reagents, except GlutenTox A1-HRP conjugated antibody, to reach room temperature (15–25°C/59–77°F) prior to testing.
- After removing multi-well test strips, reseal the aluminum bag immediately.
- The wash solution is supplied as a 10× concentrate, which must be diluted 1:10 in distilled water prior to use.
- The diluted wash solution remains stable for two weeks at 2–8°C (36–46°F).
- Each sample material should be analyzed at least in duplicate.

### Sample Preparation

Extraction of solid and semisolid samples:

- Take a representative sample of the food and mill and/or triturate it thoroughly.
- Homogenize by shaking the sample by hand for 1 min.
- Weigh 0.5 g of the sample into a test tube.
- Add 5 mL of extraction solution. Close the tube and mix vigorously using a vortex mixer for 30 s.
- Depending on the complexity of the sample matrix and whether the food sample has been processed by heat or not, follow one of the two options below.
  1. Non-heat-processed samples with simple matrix composition
    (a)Incubate the sample at room temperature (15–25°C/59–77°F) for 40 min with mild agitation (for example, using a tube rotator).
  2. Heat-processed sample and/or complex matrix composition (incurred bread)
    (a)Incubate the sample at 50°C (122°F) in a water bath for 40 min; periodically mix the sample by inverting or vortexing the tube.
- Centrifuge the suspension at 2500 × *g* for 10 min and transfer the supernatant to a clean tube.

Extraction of liquid samples:

Liquid samples without emulsions or solids do not require intensive extraction. Manual shaking or vortexing is enough, and the final step of centrifugation is not required.

- Shake the sample to homogenize.
- Add 0.5 mL of sample in a test tube.
- Add 4.5 mL of extraction solution. Close the tube and shake for 2 min manually or using a vortex mixer.

### Analysis

- Allow all reagents and test strips, except GlutenTox A1-HRP conjugated antibody, to come to room temperature (15–25°C/59–77°F) before testing.
- All assay reactions should be performed at least in duplicate.
- Prepare the appropriate dilutions of the extracted, clarified samples using the included Dilution Solution in polypropylene vials. A final volume of 300 µL is enough for the analysis of each sample. Extracted sample dilutions should be analyzed as soon as possible and any unused material discarded.
- Depending on the expected gluten content of the sample, prepare dilutions according to Table 1.
- Add 100 µL of each standard, positive control, negative control, internal control, and sample dilution to separate wells in duplicate. Cover the wells and incubate at room temperature (15–25°C/59–77°F) for 30 min.
- Eliminate the well contents by inverting the plate. Add 300 µL of diluted wash solution to all wells and incubate for 3 s. Repeat this sequence four more times for a total of five washes. Perform the washes in the same order used to load the wells in the previous step. After the last wash, invert the plate and tap it on an absorbent material such as a clean paper towel to eliminate any remaining liquid. An automatic washer is recommended for a higher reproducibility of the results.
- Add 100 µL of the GlutenTox A1-HRP conjugated antibody to each well. Cover the wells and incubate at room temperature (15–25°C/59–77°F) for 30 min.
- Wash the plate five times with 300 µL of diluted wash solution per well as indicated in the previous step.
- Add 100 µL of substrate solution to each well. Cover the wells and incubate at room temperature (15–25°C/59–77°F) for 30 min in the dark.
- Add 100 µL of stop solution to each well. Follow the same order used when adding the substrate solution in the previous step.
- Using an ELISA microplate reader with a 450 nm filter, read the absorbance (OD) of each well as soon as possible, within 30 min of the addition of stop solution.

**Table 1.** GlutenTox ELISA Rapid G12—Calibration study: dilutions according to the expected amount of gluten

		Example of dilution	
Expected amount of gluten	Dilution	Sample extract	Dilution Solution
Gluten-free (<20 ppm)	1:20	50 µL	950 µL
Low gluten (20 to 50 ppm)	1:50	20 µL	980 µL
Medium gluten (50 to 100 ppm)	1:100	10 µL	990 µL
High gluten (100 to 200 ppm)	1:200	5 µL	995 µL

### Calculations, Interpretation, and Test Result Report

- Determine average absorbance values for the replicates of each condition.
- Prepare a standard curve by plotting gliadin concentrations of each GlutenTox standard (*y*-axis) versus the respective absorbance values (*x*-axis) obtained from the calibration standards using appropriate software (for example, Excel). Please contact Hygiena Diagnóstica España to obtain an Excel template.
- Calculate the equation that defines the standard curve by second-order polynomial regression using suitable software (for instance, Excel).
- Enter the sample absorbance values obtained for each sample into this equation to obtain gliadin concentrations of the sample dilutions.
- Enter the gliadin concentration value obtained into the following formula to obtain the amount of gluten in parts per million (ppm):
  ppm gluten=(ng/mL gliadin × dilution × 2)/100
  When the absorbance (OD) of the sample is not within the values covered by the standard curve, the assay should be repeated using different dilutions.

## Validation Study

This validation study of the GlutenTox ELISA Rapid G12 test kit (Hygiena Diagnóstica España, Camas, Sevilla, Spain) for the detection and quantification of gluten in foods was conducted under the AOAC Research Institute *Performance Tested Methods*^SM^ Program: *GlutenTox ELISA Rapid G12 Kit in Select Foods Version 10, September 27, 2021* following the *Guidelines for Validation of Quantitative Gluten Methods, with Specific Examples for ELISA Assays* in conjunction with the instructions for use (INS3026) included with the test kit. Method Developer studies were conducted in the laboratory of Hygiena Diagnóstica España, S.L.U. and included calibration, spiked food matrix with all claimed matrixes, incurred food matrix with baked gluten-free bread spiked with all claimed gluten sources, bias, recovery, repeatability precision, intermediate precision (for an incurred bread matrix spiked with wheat flour), LOD, LOQ, selectivity, product consistency (from the intermediate precision), stability, and robustness testing. The independent laboratory study was conducted by Q Laboratories, Inc. (Cincinnati, OH) and included a food matrix study for corn bread and seasoning mix, both spiked with wheat flour, and an incurred matrix study for baked gluten-free bread spiked with wheat, barley, and rye flour, separately.

The AOAC OMA **2012.01** method (5) was used to prescreen natural contamination of all matrixes and to verify homogeneity on every food matrix high-level concentration stock.

### Reference Materials

Materials used for contamination (wheat, barley, and rye flours) were procured from a retail source and were independently characterized for total protein content.

Wheat flour: Gallo (Carrefour Supermarket, Camas, Sevilla, Spain); Batch L71 018 11
Rye flour: Barry Farm (The Barry Farm, Wapakoneta, OH); Batch 90010
Barley flour (whole grain): Marhaba (Kalustyan’s, NY)

- *Wheat flour.*—The wheat flour was tested by the Kjeldahl nitrogen method, obtaining 9.13% protein (9.13 g of protein in 100 g of sample). This value was multiplied by 0.80 (the conversion factor of wheat), resulting in 7.3% gluten (7.3 g of gluten protein in 100 g of sample). This percent value was then multiplied by 10 000 to estimate the mg/kg value at 73 000.
  This is equivalent to 73 mg of gluten per gram of flour.
- *Barley flour.*—The barley flour was tested by the Kjeldahl nitrogen method, obtaining 9.94% protein (9.94 g of protein in 100 g of sample). This value was multiplied by 0.75 (the conversion factor of barley), resulting in 7.46% gluten (7.46 g of gluten protein in 100 g of sample). This percent value was then multiplied by 10 000 to estimate the mg/kg value at 74 600.
  This is equivalent to 74.6 mg of gluten per gram of flour.
- *Rye flour.*—The rye flour was tested by the Kjeldahl nitrogen method, obtaining 8.46% protein (8.46 g of protein in 100 g of sample). This value was multiplied by 0.48 (the conversion factor of rye), resulting in 4.06% gluten (4.06 g of gluten protein in 100 g of sample). This percent value was then multiplied by 10 000 to estimate the mg/kg value at 40 600.
  This is equivalent to 40.6 mg of gluten per gram of flour.
- *Preparation of high-level gluten source flour concentration stocks.*—A 75–200 mg/kg (ppm) stock mixture was created by blending the gluten source flour into the matrix material (total weight of 500 g). This mixture was very thoroughly blended to ensure homogeneity.

#### Preparation of Validation Materials

##### Preparation of High-Level Wheat Flour Concentration Stocks for Dry Matrix Spiking and Test Portions

A high-level concentration stock of gluten from wheat flour (75–200 mg/kg gluten) was prepared in each food matrix.

All matrixes were prescreened using the AOAC OMA **2012.01** method (5) to detect natural contamination prior to the study startup.

Matrixes for spiking were prepared by chopping, grinding, and mixing until they were finely ground and uniform.

From the bulk matrix, 500 g of each gluten-free food matrix was weighed on an analytical balance and added into the bowl mixer (Cecotec Mixer). The spiking reference material needed (from wheat flour) was weighed and slowly added to the gluten-free food matrix in the bowl mixer mixing by hand, thoroughly and constantly, until the mixture appeared fully incorporated. Then, the content of the bowl was first mixed in the mixer at maximum power (1000 w) for 5 min and, subsequently, mixed manually with a spoon for 5 min. This mixing process was repeated four times to achieve a homogeneous distribution of the contaminant within the food matrix. About 20 g of the mixture was aliquoted in a 125 mL polypropylene flat-bottom tube (the rest of the mixture was stored in a self-closing plastic bag in a dry place).

Homogeneity testing on the high concentration stocks was performed in every food matrix stock by analyzing 10 test portions randomly chosen using the AOAC OMA **2012.01** method (5).

#### Preparation of Dry Test Portions

The high-level wheat flour concentration stocks were diluted, using a stepwise dilution scheme, to prepare gluten-contaminated matrix batches for each level being tested (0, 5, 10, and 20 ppm of gluten from wheat flour). Each contaminated matrix batch went through the same mixing process (as the high concentration stocks) four times.

A portion of 500 g of the 20 mg/kg gluten concentration was prepared for each gluten-free matrix using the finely ground and uniform matrix with the corresponding amount of high-level wheat flour concentration stock.

The 10 mg/kg gluten concentration was prepared for each gluten-free matrix mixing 250 g of finely ground and uniform matrix with 250 g of the corresponding matrix contaminated at 20 mg/kg of gluten.

The 5 mg/kg gluten concentration was prepared for each gluten-free matrix mixing 250 g of finely ground and uniform matrix with 250 g of the corresponding matrix contaminated at 10 mg/kg of gluten.

Six individual 0.5 g test portions of every matrix at each concentration of gluten (0, 5, 10, and 20 mg/kg of gluten from wheat flour) were portioned out into 50 mL polypropylene flat-bottom test tubes. Once prepared, the test portions were held at room temperature in a dry place until use.

##### Preparation of High-Level Wheat Flour Concentration Stocks for Moist Matrix Spiking and Test Portions

A high concentration stock of gluten from wheat flour (128.5 mg/kg gluten) was prepared for the evaporated milk matrix.

This matrix was prescreened using the AOAC OMA **2012.01** method (5) to detect natural contamination prior to the study startup.

The evaporated milk matrix for spiking was prepared by mixing until it was uniform.

Using the candidate method extraction buffer, 90 mL of the high concentration suspension stock of gluten (4000 mg/kg gluten) from wheat flour (3.755 g) was prepared. This suspension was used to spike into the gluten-free evaporated milk matrix.

Thus, 15 mL of the high concentration suspension stock of gluten was slowly added to 485 mL of the gluten-free evaporated milk matrix in a bottle, mixing thoroughly and constantly with a magnetic stirrer for 30 min until the mixture (128.5 mg/kg gluten) appeared fully incorporated. By this mixing process, a homogeneous distribution of the contaminant within the food matrix was achieved. The mixture was stored refrigerated at 4°C.

Homogeneity testing on the high concentration stock was performed in the evaporated milk matrix stock by analyzing 10 test portions randomly chosen using the AOAC OMA **2012.01** (5).

#### Preparation of Moist Test Portions

The high-level wheat flour concentration stock, prepared in gluten-free evaporated milk matrix, was diluted, using a stepwise dilution scheme, to prepare a gluten-contaminated matrix batch for each level being tested (0, 5, 10, and 20 mg/kg of gluten from wheat flour). The contaminated matrix batch went through the same mixing process (as the high concentration stocks) for 30 min.

A portion of 500 mL of the 20 mg/kg gluten concentration of evaporated milk matrix was prepared mixing the gluten-free evaporated milk matrix with the corresponding amount of high-level wheat flour concentration extract stock.

The 10 mg/kg gluten concentration level of evaporated milk matrix was prepared mixing 250 mL of the uniform gluten free evaporated milk matrix with 250 mL of the high-level wheat flour concentration extract stock contaminated at 20 mg/kg of gluten.

The 5 mg/kg gluten concentration level of evaporated milk matrix was prepared mixing 250 mL of the uniform gluten-free evaporated milk matrix with 250 mL of the high-level wheat flour concentration extract stock contaminated at 10 mg/kg of gluten.

Six individual 0.5 mL test portions of the evaporated milk matrix batch at each concentration of gluten (0, 5, 10, and 20 mg/kg of gluten from wheat flour) were portioned out into 50 mL polypropylene flat-bottom test tubes. Once prepared, the test portions were refrigerated (4°C) until use.

#### Preparation of Gluten-Free Bread and Incurred Breads by the Independent Laboratory

A bread maker was used to make and cook the gluten-free bread and the incurred breads, and a dehydrator to remove the water of the final product:

Bread maker: Bread Maker Homemade Deluxe Princess
Food dehydrator: Food Dryer Proficook PC-DR 1116

To prepare the breads, the instructions of gluten-free bread mix below were followed:

*Ingredients:*

Gluten-free bread mix: 410 g
Bakery yeast: 3.5 g
Distilled water: 330 mL
Sunflower oil: 50 mL
Spiking material: adequate amount

To calculate the amount of spiking material, the final weight of the product as the sum of gluten-free bread mix weight (410 g) and sunflower oil volume (50 mL∼ 50 g), giving a total of 460 g, was considered.

*Procedure:*

1. 205 g gluten-free bread mix was added to the bread pan.
2. The spiking material was added to the bread pan, spreading it over the blank material.
3. Liquid ingredients (50 mL sunflower oil and 330 mL distilled water) were added to the bread pan.
4. 205 g gluten-free bread mix was added to the bread pan.
5. 3.5 g bakery yeast was added to the bread pan.

To cook the bread mass, the specific program for gluten-free bread offered by the bread maker was followed.

Once the baking process was finished, the breads were cut into small pieces and frozen at −20°C overnight.

The following day, the material was ground while it was still frozen. Afterwards, the material was dehydrated for 48 h at 70°C using the food dehydrator.

After the dehydration process, the material was ground again and mixed using a food mixer.

## Results

### Calibration Study

The calibration study was conducted in every extracted sample dilution (extracts prepared directly from wheat flour using the candidate method extraction mentioned in the GlutenTox ELISA Rapid G12 manual (Table 1) according to the expected amount of gluten.

Using the set of standards/calibrators supplied with the kit, a dose response curve was determined at every extracted sample dilution, using five (or six) concentrations over the concentration range of the kit with five replicates each.

For the 1:20, 1:50, and 1:100 dilutions, five concentrations of the extracted samples (0, 2, 5, 10, and 15 mg/kg of gluten), (0, 5, 10, 20, and 35 mg/kg of gluten), and (0, 10, 20, 50, and 70 mg/kg of gluten) were tested, respectively.

For the 1:200 dilution, six concentrations of the extracted samples (0, 10, 20, 50, 70, and 100 mg/kg of gluten) were also tested.

The results are shown in Figures 1–8.

**Figure 1.**
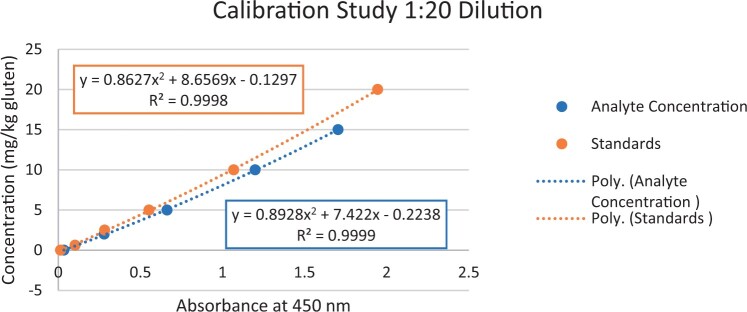
GlutenTox ELISA Rapid G12—Calibration study: 1:20 dilution.

**Figure 2.**
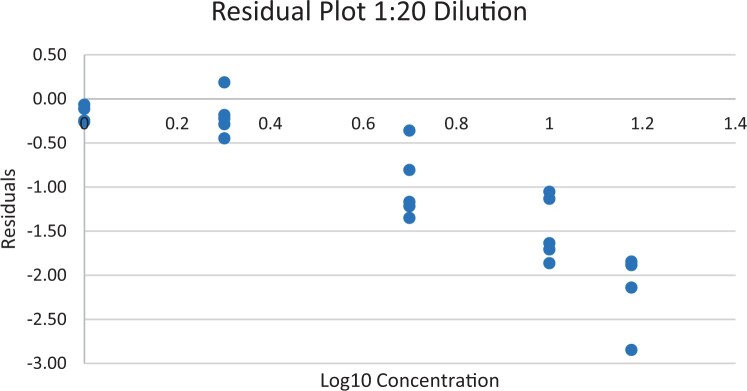
GlutenTox ELISA Rapid G12—Calibration study: Residual plot 1:20 dilution.

**Figure 3.**
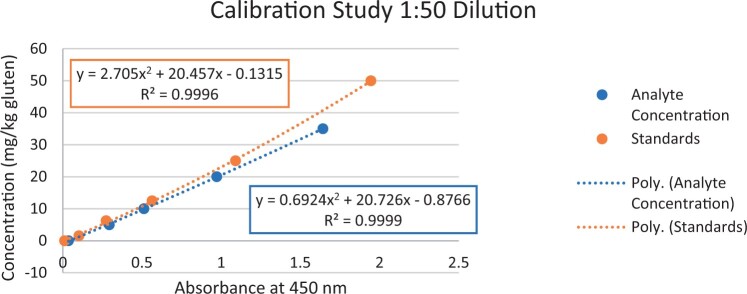
GlutenTox ELISA Rapid G12—Calibration study: 1:50 dilution.

**Figure 4.**
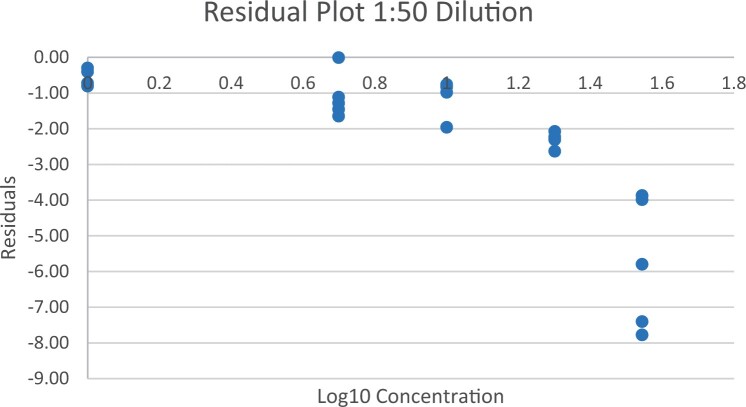
GlutenTox ELISA Rapid G12—Calibration study: Residual plot 1:50 dilution.

**Figure 5.**
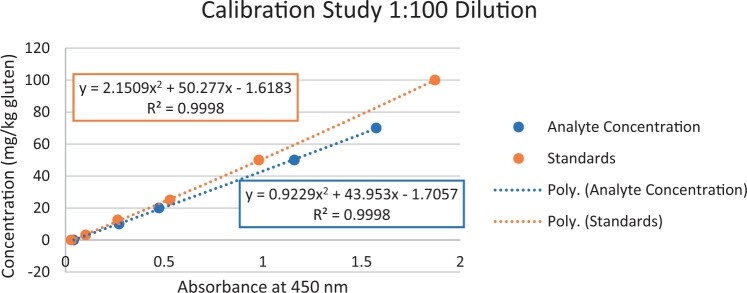
GlutenTox ELISA Rapid G12—Calibration study: 1:100 dilution.

**Figure 6.**
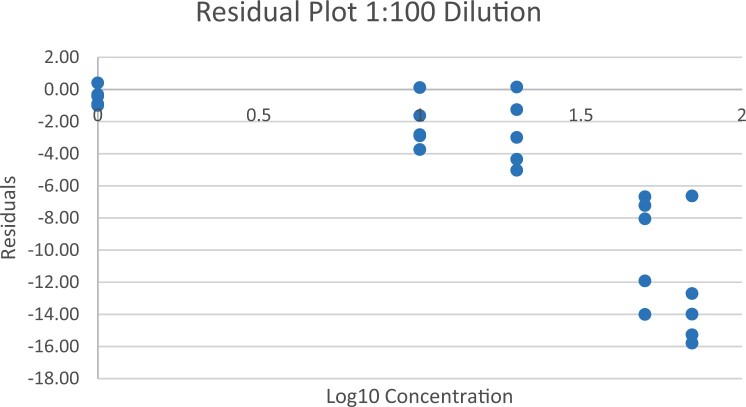
GlutenTox ELISA Rapid G12—Calibration study: Residual plot 1:100 dilution.

**Figure 7.**
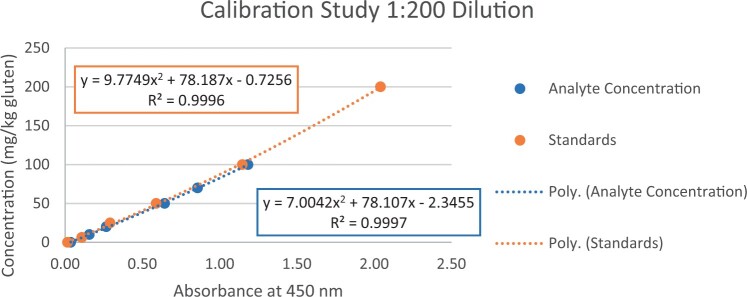
GlutenTox ELISA Rapid G12—Calibration study: 1:200 dilution.

**Figure 8.**
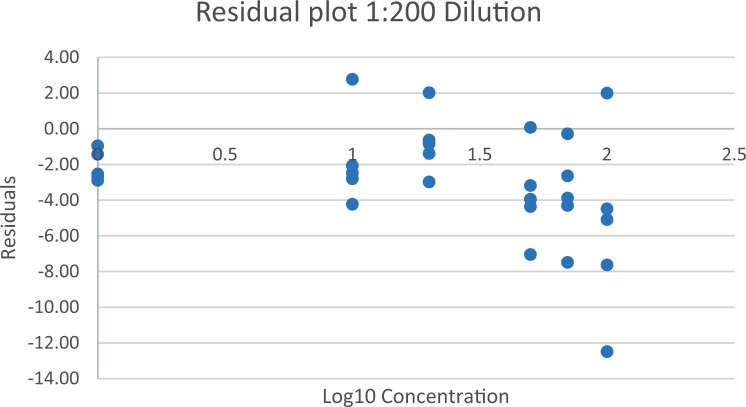
GlutenTox ELISA Rapid G12—Calibration study: Residual plot 1:200 dilution.

The calibration study conducted with the GlutenTox ELISA Rapid G12 in all dilutions produced results where a trend of a nonrandom pattern was found in the higher analyte concentrations of each dilution.

### Matrix Study With Wheat Flour (Non-Processed Foods)

The matrix study was performed to test the ability of the GlutenTox ELISA Rapid G12 test kit to determine the gluten-containing reference material (wheat flour) in each of the five selected food matrixes (soy flour, corn bread/biscuit, rolled oat, seasoning mix, and evaporated milk). Spikes were tested at four different levels of gluten (0, 5, 10, and 20 mg/kg). For each spike level, six blind-coded (individually extracted) test portions were analyzed by one technician using the GlutenTox ELISA Rapid G12 method. Test portions (replicates) were taken from a single lot of bulk material. This lot of bulk material of every matrix at each spike level was prepared by using a stepwise dilution scheme, diluting the high concentration stock previously contaminated with wheat flour. All matrixes were prescreened using the AOAC OMA **2012.01** (5) to detect natural contamination prior to the study startup.

The matrix study determines the bias, recovery, repeatability precision, LOD, and LOQ of the GlutenTox ELISA Rapid G12 test kit.

**Figure 9.**
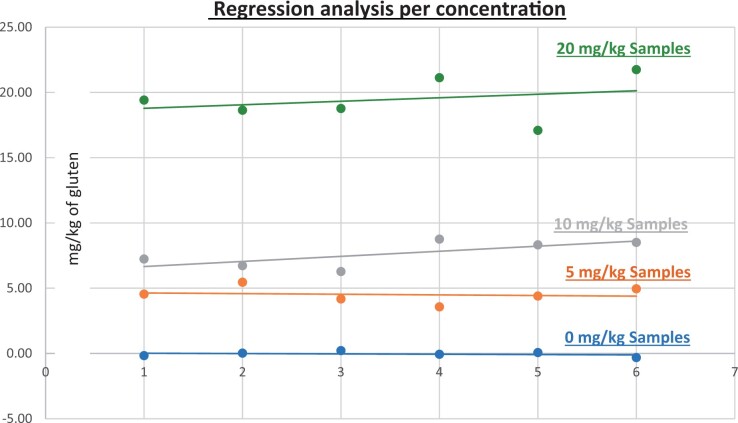
GlutenTox ELISA Rapid G12—Matrix study with wheat flour: Soy flour (Regression analysis per concentration).

**Figure 10.**
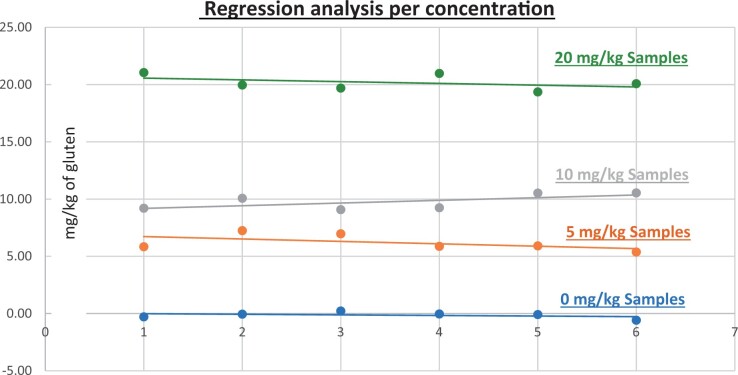
GlutenTox ELISA Rapid G12—Matrix study with wheat flour: Corn bread (Regression analysis per concentration).

**Figure 11.**
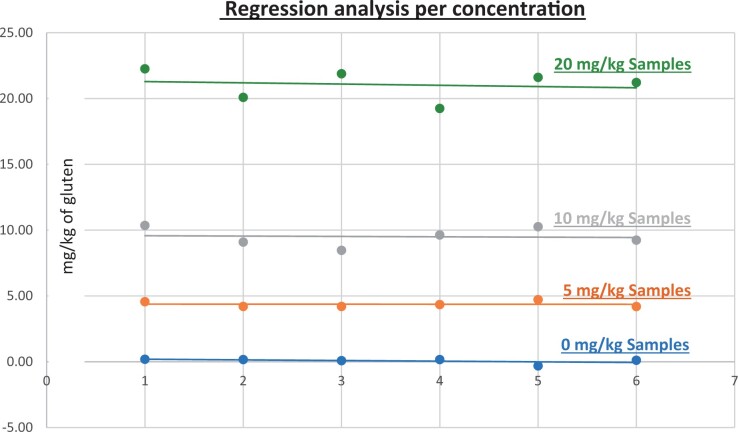
GlutenTox ELISA Rapid G12—Matrix study with wheat flour: Seasoning mix (Regression analysis per concentration).

**Figure 12.**
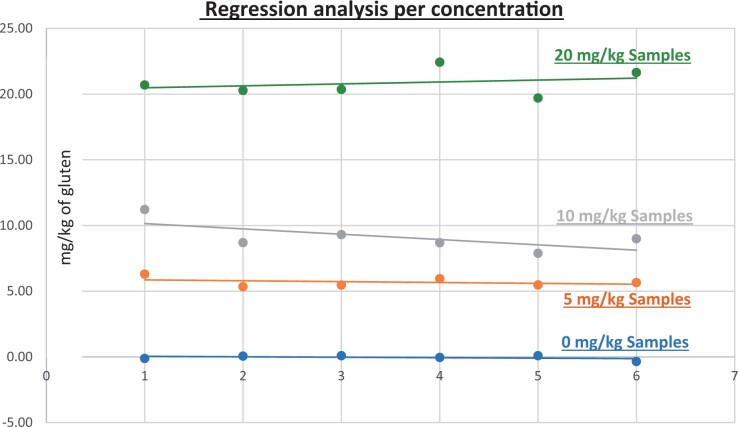
GlutenTox ELISA Rapid G12—Matrix study with wheat flour: Rolled oats (Regression analysis per concentration).

**Figure 13.**
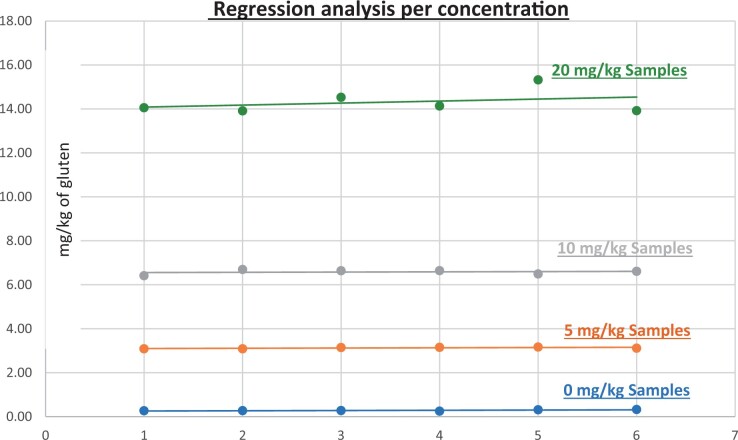
GlutenTox ELISA Rapid G12—Matrix study with wheat flour: Evaporated milk (Regression analysis per concentration).

- *Soy flour.—*The evaluation of sample extracts for recovery produced average values of <LOQ recovery at 0 mg/kg spike level of gluten, 90.25% recovery (4.51 mg/kg mean value) at 5 mg/kg spike level, 76.32% recovery (7.63 mg/kg mean value) at 10 mg/kg spike level, and 97.30% recovery (19.46 mg/kg mean value) at 20 mg/kg spike level.
  The evaluation of the same sample extracts for repeatability produced average values of 14.36% RSD at 5.0 mg/kg, 13.52% RSD at 10 mg/kg, and 8.83% RSD at 20 mg/kg spike level of gluten.
  For soy flour, a summary of results is presented in Table 2 and Figure 9.
- *Corn bread.—*The evaluation of samples extracts for recovery produced average values of <LOQ recovery at 0 mg/kg spike level of gluten, 123.95% recovery (6.20 mg/kg mean value) at 5 mg/kg spike level, 97.70% recovery (9.77 mg/kg mean value) at 10 mg/kg spike level, and 100.91% recovery (20.18 mg/kg mean value) at 20 mg/kg spike level.
  The evaluation of the same sample extracts for repeatability produced average values of 11.79% RSD at 5.0 mg/kg, 7.00% RSD at 10 mg/kg, and 3.41% RSD at 20 mg/kg spike level of gluten.
  For corn bread, a summary of results is presented in Table 2 and Figure 10.
- *Seasoning mix.—*The evaluation of samples extracts for recovery produced average values of <LOQ recovery at 0 mg/kg spike level of gluten, 87.39% recovery (4.37 mg/kg mean value) at 5 mg/kg spike level, 95.08% recovery (9.51 mg/kg mean value) at 10 mg/kg spike level, and 105.25% recovery (21.05 mg/kg mean value) at 20 mg/kg spike level.
  The evaluation of the same sample extracts for repeatability produced average values of 5.10% RSD at 5.0 mg/kg, 7.63% RSD at 10 mg/kg, and 5.49% RSD at 20 mg/kg spike level of gluten.
  For seasoning mix, a summary of results is presented in Table 2 and Figure 11.
- *Rolled oats.—*The evaluation of samples extracts for recovery produced average values of <LOQ recovery at 0 mg/kg spike level of gluten, 113.96% recovery (5.70 mg/kg mean value) at 5 mg/kg spike level, 91.31% recovery (9.13 mg/kg mean value) at 10 mg/kg spike level, and 104.26% recovery (20.85 mg/kg mean value) at 20 mg/kg spike level.
  The evaluation of the same sample extracts for repeatability produced average values of 6.31% RSD at 5.0 mg/kg, 12.33% RSD at 10 mg/kg, and 4.82% RSD at 20 mg/kg spike level of gluten.
  For rolled outs, a summary of results is presented in Table 2 and Figure 12.
- *Evaporated milk.—*The evaluation of samples extracts for recovery produced average values of <LOQ recovery at 0 mg/kg spike level of gluten, 63.00% recovery (3.13 mg/kg mean value) at 5 mg/kg spike level, 66.00% recovery (6.58 mg/kg mean value) at 10 mg/kg spike level, and 72.00% recovery (14.31 mg/kg mean value) at 20 mg/kg spike level.
  The evaluation of the same sample extracts for repeatability produced average values of 1.00% RSD at 5.0 mg/kg, 2.00% RSD at 10 mg/kg, and 4.00% RSD at 20 mg/kg spike level of gluten.
  For evaporated milk, a summary of results is presented in Table 2 and Figure 13.

**Table 2.** GlutenTox ELISA Rapid G12—Food matrix study with wheat flour from candidate and from independent laboratory (analyst 1 and analyst 2)

		Candidate				
Matrix	Target contamination level, mg/kg	Mean concentration obtained, mg/kg	Recovery	Bias	S_r_	RSD_r_
Soy flour	0	−0.05	<LOD	−0.05	0.187	−410.941
	5	4.51	90.25	−0.49	0.648	14.361
	10	7.63	76.32	−2.37	1.031	13.519
	20	19.46	97.30	−0.54	1.719	8.833
Corn bread	0	−0.14	<LOD	−0.14	0.271	−190.988
	5	6.20	123.95	1.20	0.730	11.793
	10	9.77	97.70	−0.23	0.684	7.001
	20	20.18	100.91	0.18	0.687	3.408
Seasoning mix	0	0.07	<LOD	0.07	0.190	280.276
	5	4.37	87.39	−0.63	0.222	5.099
	10	9.51	95.08	−0.49	0.725	7.635
	20	21.05	105.25	1.05	1.154	5.486
Rolled oats	0	−0.05	<LOD	−0.05	0.170	−370.012
	5	5.70	113.96	0.70	0.359	6.307
	10	9.13	91.31	−0.87	1.126	12.333
	20	20.85	104.26	0.85	1.005	4.822
Evaporated milk	0	0.28	<LOD	0.28	0.029	10.294
	5	3.13	62.54	−1.87	0.035	1.107
	10	6.58	65.82	−3.42	0.107	1.624
	20	14.31	71.56	−5.69	0.543	3.796

		Independent laboratory, analyst 1				
Matrix	Target contamination level, mg/kg	Mean concentration obtained, mg/kg	Recovery	Bias	S_r_	RSD_r_
Corn bread	0	0.328	/	0.328	0.015	4.482
	5	4.755	95.10	−0.245	0.520	10.938
	10	7.643	76.43	−2.357	0.570	7.452
	20	20.877	104.39	0.877	2.473	11.844
Seasoning mix	0	0.197	/	0.197	0.029	14.638
	5	3.979	79.589	−1.021	0.182	4.582
	10	11.578	115.780	1.578	0.369	3.189
	20	21.268	106.340	1.268	1.320	6.206

		Independent laboratory, analyst 2				
Matrix	Target contamination level, mg/kg	Mean concentration obtained, mg/kg	Recovery	Bias	S_r_	RSD_r_
Corn bread	0	0.293	/	0.293	0.029	9.814
	5	4.683	93.667	−0.317	0.182	3.894
	10	7.561	75.617	−2.438	0.566	7.486
	20	20.916	104.580	0.916	2.431	11.623
Seasoning mix	0	0.163	/	0.163	0.035	21.443
	5	4.165	78.833	−1.073	0.164	4.165
	10	11.452	114.517	1.452	0.314	2.739
	20	20.984	104.922	0.984	1.559	7.430

### Matrix Study With Barley and Rye Flours (Non-Processed Foods)

The matrix study was performed to test the ability of the GlutenTox ELISA Rapid G12 test kit to determine the gluten-containing reference material (barley and rye flour) in each of the five selected food matrixes (soy flour, corn bread/biscuit, rolled oat, seasoning mix, and evaporated milk). Spikes were tested at three different levels of gluten (0, 10, and 20 mg/kg). For each spike level, five blind-coded (individually extracted replicate) test portions were analyzed by one technician using the GlutenTox ELISA Rapid G12 method. Matrixes were prepared and contaminated in the same manner as the wheat flour spiked test portions, using the appropriate conversion factors for barley (0.75) and rye (0.48).

The matrix study determines the bias, recovery, and repeatability precision of the GlutenTox ELISA Rapid G12 test kit.

**Figure 14.**
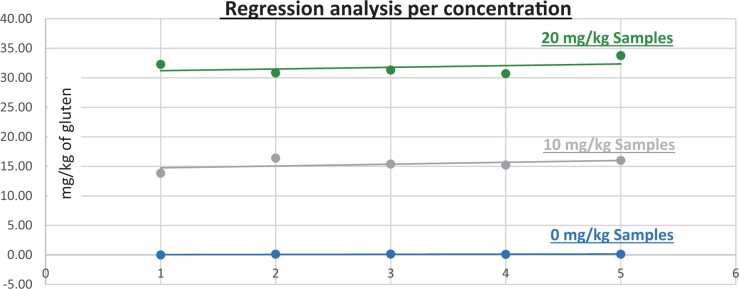
GlutenTox ELISA Rapid G12—Matrix study with barley flour: Soy flour (Regression analysis per concentration).

**Figure 15.**
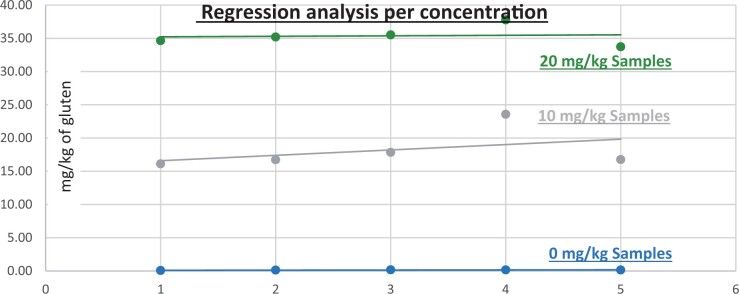
GlutenTox ELISA Rapid G12—Matrix study with rye flour: Soy flour (Regression analysis per concentration).

**Figure 16.**
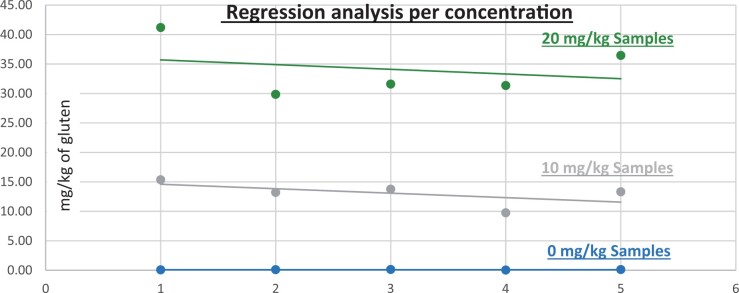
GlutenTox ELISA Rapid G12—Matrix study with barley flour: Corn bread (Regression analysis per concentration).

**Figure 17.**
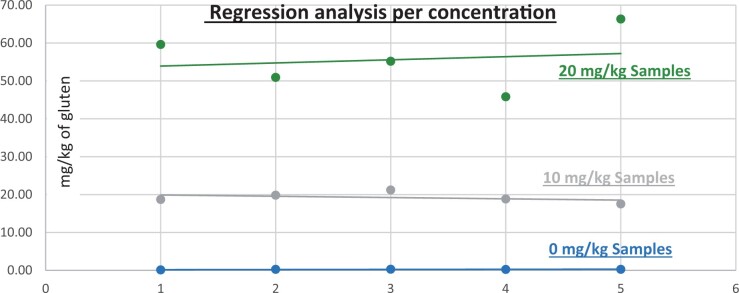
GlutenTox ELISA Rapid G12—Matrix study with rye flour: Corn bread (Regression analysis per concentration).

**Figure 18.**
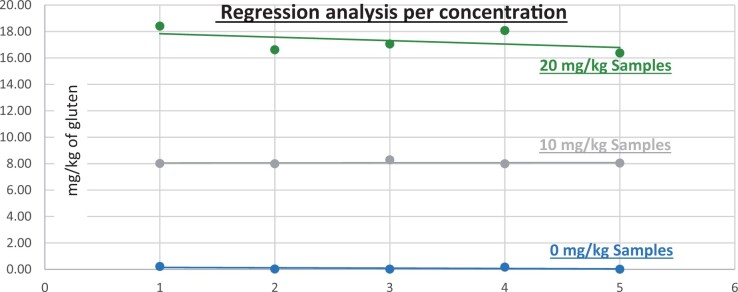
GlutenTox ELISA Rapid G12—Matrix study with barley flour: Seasoning mix (Regression analysis per concentration).

**Figure 19.**
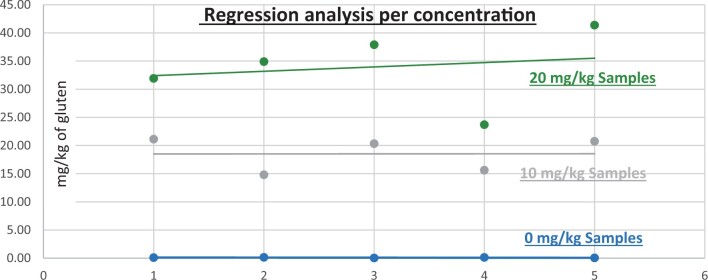
GlutenTox ELISA Rapid G12—Matrix study with rye flour: Seasoning mix (Regression analysis per concentration).

**Figure 20.**
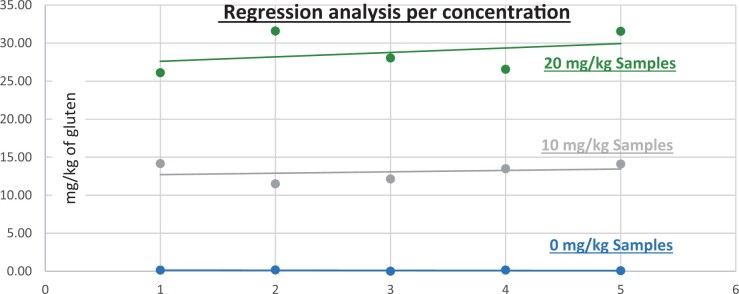
GlutenTox ELISA Rapid G12—Matrix study with barley flour: Rolled oats (Regression analysis per concentration).

**Figure 21.**
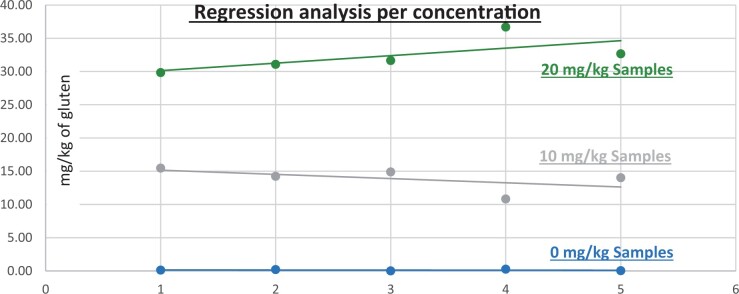
GlutenTox ELISA Rapid G12—Matrix study with rye flour: Rolled oats (Regression analysis per concentration).

**Figure 22.**
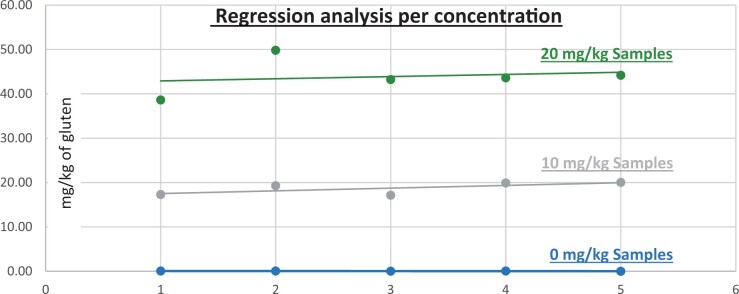
GlutenTox ELISA Rapid G12—Matrix study with barley flour: Evaporated milk (Regression analysis per concentration).

**Figure 23.**
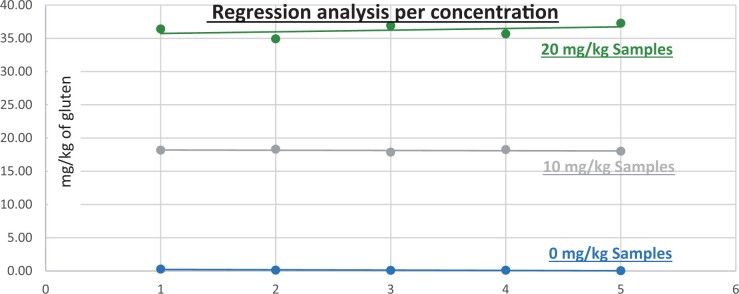
GlutenTox ELISA Rapid G12—Matrix study with rye flour: Evaporated milk (Regression analysis per concentration).

- *Soy flour.—*The evaluation of samples extracts for recovery produced average values of <LOQ recovery at 0 mg/kg spike level of gluten, 153.736% recovery (15.374 mg/kg mean value) at 10 mg/kg spike level gluten from barley and 182.064% recovery (18.206 mg/kg mean value) at 10 mg/kg spike level gluten from rye, 158.897% recovery (31.779 mg/kg mean value) at 20 mg/kg spike level gluten from barley and 176.878% recovery (35.376 mg/kg mean value) at 20 mg/kg spike level gluten from rye.
  The evaluation of the same sample extracts for repeatability produced average values of 6.359% RSD at 10 mg/kg, and 4.014% RSD at 20 mg/kg spike level of gluten from barley and 16.829% RSD at 10 mg/kg, and 4.244% RSD at 20 mg/kg spike level of gluten from rye.
  For soy flour, a summary of results is presented in Table 3 and Figures 14 and 15.
- *Corn bread.—*The evaluation of samples extracts for recovery produced average values of <LOQ recovery at 0 mg/kg spike level of gluten, 130.83% recovery (13.08 mg/kg mean value) at 10 mg/kg spike level gluten from barley and 192.199% recovery (19.220 mg/kg mean value) at 10 mg/kg spike level gluten from rye, 170.484% recovery (34.097 mg/kg mean value) at 20 mg/kg spike level gluten from barley and 277.861% recovery (55.572 mg/kg mean value) at 20 mg/kg spike level gluten from rye.
  The evaluation of the same sample extracts for repeatability produced average values of 15.675% RSD at 10 mg/kg, and 13.738% RSD at 20 mg/kg spike level of gluten from barley and 7.171% RSD at 10 mg/kg, and 14.206% RSD at 20 mg/kg spike level of gluten from rye.
  For corn bread, a summary of results is presented in Table 3 and Figures 16 and 17.
- *Seasoning mix.—*The evaluation of samples extracts for recovery produced average values of <LOQ recovery at 0 mg/kg spike level of gluten, 80.674% recovery (8.067 mg/kg mean value) at 10 mg/kg spike level gluten from barley and 185.243% recovery (18.524 mg/kg mean value) at 10 mg/kg spike level gluten from rye, 86.534% recovery (17.307 mg/kg mean value) at 20 mg/kg spike level gluten from barley, and 169.806% recovery (33.961 mg/kg mean value) at 20 mg/kg spike level gluten from rye.
  The evaluation of the same sample extracts for repeatability produced average values of 1.529% RSD at 10 mg/kg, and 5.176% RSD at 20 mg/kg spike level of gluten from barley and 16.484% RSD at 10 mg/kg, and 19.797% RSD at 20 mg/kg spike level of gluten from rye.
  For seasoning mix, a summary of results is presented in Table 3 and Figures 18 and 19.
- *Rolled oats.—*The evaluation of samples extracts for recovery produced average values of <LOQ recovery at 0 mg/kg spike level of gluten, 130.760% recovery (13.076 mg/kg mean value) at 10 mg/kg spike level gluten from barley and 138.901% recovery (13.890 mg/kg mean value) at 10 mg/kg spike level gluten from rye, 143.880% recovery (28.776 mg/kg mean value) at 20 mg/kg spike level gluten from barley and 161.912% recovery (32.382 mg/kg mean value) at 20 mg/kg spike level gluten from rye.
  The evaluation of the same sample extracts for repeatability produced average values of 9.162% RSD at 10 mg/kg, and 9.231% RSD at 20 mg/kg spike level of gluten from barley and 12.977% RSD at 10 mg/kg, and 8.069% RSD at 20 mg/kg spike level of gluten from rye.
  For rolled oats, a summary of results is presented in Table 3 and Figures 20 and 21.
- *Evaporated milk.—*The evaluation of samples extracts for recovery produced average values of <LOQ recovery at 0 mg/kg spike level of gluten, 187.370% recovery (18.737 mg/kg mean value) at 10 mg/kg spike level gluten from barley and 181.292% recovery (18.129 mg/kg mean value) at 10 mg/kg spike level gluten from rye, 219.441% recovery (43.888 mg/kg mean value) at 20 mg/kg spike level gluten from barley and 181.157% recovery (36.231 mg/kg mean value) at 20 mg/kg spike level gluten from rye.
  The evaluation of the same sample extracts for repeatability produced average values of 7.516% RSD at 10 mg/kg, and 9.071% RSD at 20 mg/kg spike level of gluten from barley and 1.008% RSD at 10 mg/kg, and 2.628% RSD at 20 mg/kg spike level of gluten from rye.
  For evaporated milk, a summary of results is presented in Table 3 and Figures 22 and 23.

**Table 3.** GlutenTox ELISA Rapid G12—Food matrix study with barley and rye flours from candidate

		Candidate				
Matrix/Contaminant	Target contamination level, mg/kg	Mean concentration obtained, mg/kg	Recovery	Bias	S_r_	RSD_r_
Soy flour Barley flour	0	0.098	<LOD	0.098	0.059	60.345
	10	15.374	153.736	5.374	0.978	6.359
	20	31.779	158.897	11.779	1.276	4.014
Corn bread Barley flour	0	0.098	<LOD	0.098	0.041	41.214
	10	13.083	130.832	3.083	2.051	15.675
	20	34.097	170.484	14.097	4.684	13.738
Seasoning mix Barley flour	0	0.093	<LOD	0.093	0.098	105.809
	10	8.067	80.674	−1.933	0.123	1.529
	20	17.307	86.534	−2.693	0.896	5.176
Rolled oats Barley flour	0	0.114	<LOD	0.114	0.068	59.686
	10	13.076	130.760	3.076	1.198	9.162
	20	28.776	143.880	8.776	2.656	9.231
Evaporated milk Barley flour	0	0.039	<LOD	0.039	0.023	59.237
	10	18.737	187.370	8.737	1.408	7.516
	20	43.888	219.441	23.888	3.981	9.071

		Candidate				
Matrix/Contaminant	Target contamination level, mg/kg	Mean concentration obtained, mg/kg	Recovery	Bias	S_r_	RSD_r_
Soy flour Rye flour	0	0.138	<LOD	0.138	0.040	28.893
	10	18.206	182.064	8.206	3.064	16.829
	20	35.376	176.878	15.376	1.501	4.244
Corn bread Rye flour	0	0.234	<LOD	0.234	0.074	31.512
	10	19.220	192.199	9.220	1.378	7.171
	20	55.572	277.861	35.572	7.895	14.206
Seasoning mix Rye flour	0	0.086	<LOD	0.086	0.054	62.831
	10	18.524	185.243	8.524	3.054	16.484
	20	33.961	169.806	13.961	6.723	19.797
Rolled oats Rye flour	0	0.125	<LOD	0.125	0.107	86.002
	10	13.890	138.901	3.890	1.803	12.977
	20	32.382	161.912	12.382	2.613	8.069
Evaporated milk Rye flour	0	0.125	<LOD	0.125	0.095	76.082
	10	18.129	181.292	8.129	0.183	1.008
	20	36.231	181.157	16.231	0.952	2.628

**Figure 24.**
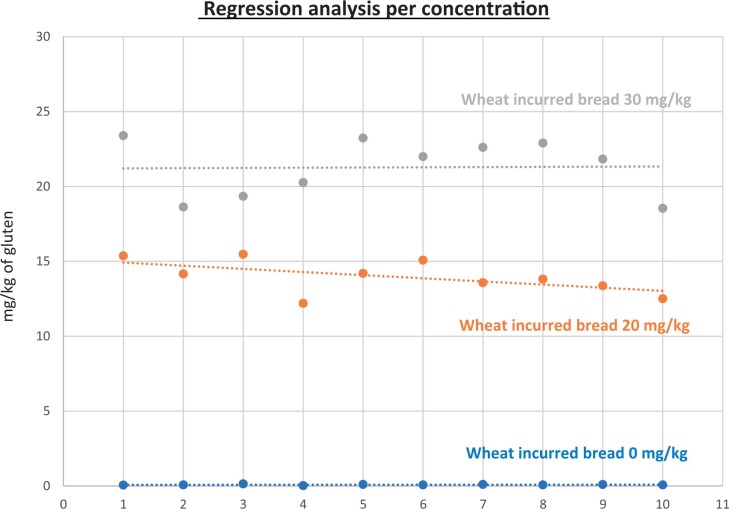
GlutenTox ELISA Rapid G12—Incurred matrix study with wheat flour: Regression analysis per concentration.

**Figure 25.**
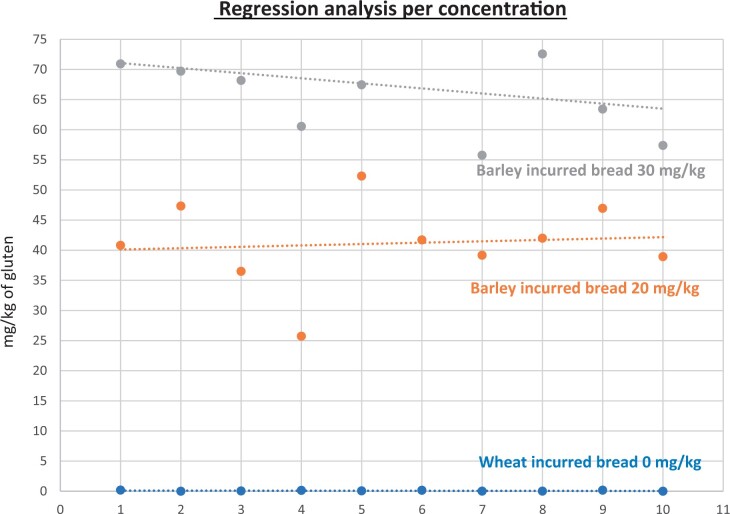
GlutenTox ELISA Rapid G12—Incurred matrix study with barley flour: Regression analysis per concentration.

**Figure 26.**
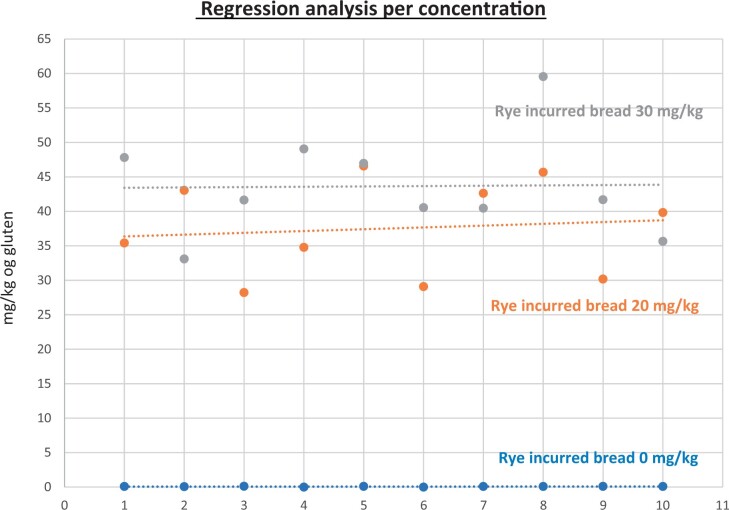
GlutenTox ELISA Rapid G12—Incurred matrix study with rye flour: Regression analysis per concentration.

**Figure 27.**
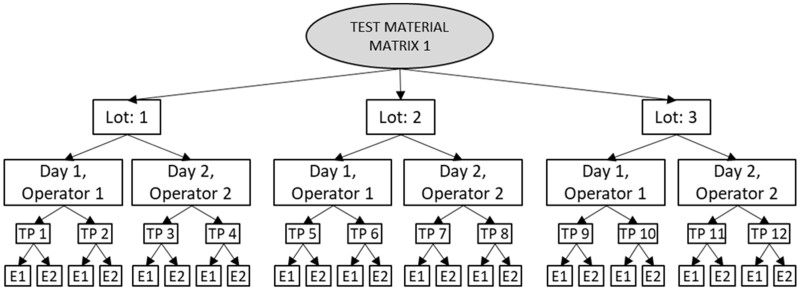
Design 2b. Lot: test kit lot, TP: test portion, E: ELISA measurement. Design 2b can be used to estimate intermediate precision, repeatability, ELISA variance, and lot-to-lot product consistency.

### Incurred Matrix Testing With Wheat, Barley, and Rye Flours (Processed Foods)

The incurred matrix study was conducted in the same fashion as the food matrix study but only on bread (gluten-free bread mix).

Incurred bread matrixes were spiked, baked, and prepared by the independent laboratory. The uncooked matrixes were spiked at three concentrations of gluten (0, 20, and 30 mg/kg) with the target analyte (from wheat flour, barley flour, or rye flour) and then baked/cooked. The independent laboratory prepared the test portions for testing at the independent laboratory and Hygiena’s laboratory to ensure both internal and independent validation studies were conducted using the same incurred test portions.

The GlutenTox ELISA Rapid G12 method was tested with 10 blank incurred test portions and 20 incurred test portions spiked at 20 mg/kg gluten (10 replicates) and 30 mg/kg gluten (10 replicates) with wheat flour. The same procedure was carried out when the target analyte was from barley flour or rye flour.

The results are shown in Table 4 and Figures 24, 25, and 26.

**Table 4.** GlutenTox ELISA Rapid G12—Incurred matrix study from candidate and from independent laboratory (analyst 1 and analyst 2)

		Candidate				
Matrix/Contaminant	Target contamination level, mg/kg	Mean concentration obtained, mg/kg	Recovery	Bias	S_r_	RSD_r_
Baked bread Wheat flour	0	0.084	<LOD	0.084	0.033	39.11
	20	13.976	69.882	−6.024	1.125	8.047
	30	21.276	70.921	−8.724	1.909	8.974
Baked bread Barley flour	0	0.080	<LOD	0.080	0.077	95.75
	20	41.143	205.717	21.143	7.191	17.478
	30	67.282	224.273	37.282	8.953	13.307
Baked bread Rye flour	0	0.078	<LOD	0.078	0.041	52.89
	20	37.540	187.699	17.540	6.943	18.494
	30	43.646	145.486	13.646	7.553	17.305

		Independent laboratory, analyst 1				
Matrix/Contaminant	Target contamination level, mg/kg	Mean concentration obtained, mg/kg	Recovery	Bias	S_r_	RSD_r_
Baked bread Wheat flour	0	0.141	/	0.141	0.035	24.992
	20	10.576	52.881	−9.424	1.077	10.179
	30	16.650	55.501	−13.350	1.639	9.846
Baked bread Barley flour	0	0.141	/	0.141	0.035	24.992
	20	33.151	165.755	13.151	5.121	15.446
	30	79.338	264.460	49.338	17.988	26.673
Baked bread Rye flour	0	0.141	/	0.141	0.035	24.992
	20	27.954	139.768	7.954	5.651	20.214
	30	40.598	135.325	10.578	5.024	12.374

		Independent laboratory, analyst 2				
Matrix/Contaminant	Target contamination level, mg/kg	Mean concentration obtained, mg/kg	Recovery	Bias	S_r_	RSD_r_
Baked bread Wheat flour	0	0.096	/	0.096	0.032	32.787
	20	10.654	53.271	−9.346	1.082	10.156
	30	16.749	55.828	−13.251	1.596	9.528
Baked bread Barley flour	0	0.096	/	0.096	0.032	32.787
	20	33.385	166.927	13.385	5.189	15.543
	30	80.039	266.796	50.039	18.356	22.934
Baked bread Rye flour	0	0.096	/	0.096	0.032	32.787
	20	27.746	138.728	7.746	6.021	21.700
	30	41.013	136.709	11.013	5.109	12.457

- *Incurred matrix testing with wheat flour.—*The evaluation of incurred samples extracts for recovery produced average values of <LOQ recovery at 0 mg/kg spike level of gluten, 69.9% recovery at 20 mg/kg spike level, and 70.9% recovery at 30 mg/kg spike level.
  The evaluation of the same incurred sample extracts for repeatability produced average values of 8.05% RSD at 20 mg/kg spike level and 8.97% RSD at 30 mg/kg spike level of gluten.
- *Incurred matrix testing with barley flour.—*The evaluation of incurred samples extracts for recovery produced average values of <LOQ recovery at 0 mg/kg spike level of gluten, 205.7% recovery at 20 mg/kg spike level, and 224.2% recovery at 30 mg/kg spike level.
  The evaluation of the same incurred sample extracts for repeatability produced average values of 17.47% RSD at 20 mg/kg spike level and 13.3% RSD at 30 mg/kg spike level of gluten.
- *Incurred matrix testing with rye flour.—*The evaluation of incurred samples extracts for recovery produced average values of <LOQ recovery at 0 mg/kg spike level of gluten, 187.69% recovery at 20 mg/kg spike level, and 145.48% recovery at 30 mg/kg spike level.
  The evaluation of the same incurred sample extracts for repeatability produced average values of 18.49% RSD at 20 mg/kg spike level and 17.3% RSD at 30 mg/kg spike level of gluten.

### LOD and LOQ Determinations of Spiked Matrixes (Unprocessed)

Data are submitted showing the LOD and LOQ of one sample of each of the gluten-free matrixes (blank test portions) tested 10 times. The LOD is expressed as the mean value of the negative sample determination (blank result) plus 3.3 SDs.

LOD was determined to be 0.77 mg/kg (soy flour), 0.74 mg/kg (corn bread), 0.72 mg/kg (rolled oats), 0.77 mg/kg (seasoning mix), and 0.69 mg/kg (evaporated milk) by GlutenTox ELISA Rapid G12, with a mean LOD of 0.738 mg/kg across all matrixes tested.

The LOQ is expressed as the mean value of the negative sample determination (blank result) plus 10 SDs.

LOQ was determined to be 1.59 mg/kg (soy flour), 1.51 mg/kg (corn bread), 1.57 mg/kg (rolled oats), 1.70 mg/kg (seasoning mix), and 1.46 mg/kg (evaporated milk) by GlutenTox ELISA Rapid G12, with a mean LOQ of 1.568 mg/kg across all matrixes tested.

A summary of LOD and LOQ results is presented in Tables 5 and 6.

**Table 5.** GlutenTox ELISA Rapid G12—LOD-LOQest study

Blank matrixes: Concentration (mg/kg gluten)					
Replicate	Soy flour	Corn bread	Rolled oats	Seasoning mix	Evaporated milk
1	0.256	0.237	0.373	0.419	0.468
2	0.444	0.419	0.298	0.174	0.241
3	0.453	0.481	0.390	0.381	0.392
4	0.319	0.281	0.022	0.423	0.121
5	0.436	0.499	0.369	0.219	0.361
6	0.104	0.231	0.361	0.436	0.348
7	0.440	0.445	0.161	0.407	0.263
8	0.478	0.423	0.407	0.306	0.348
9	0.256	0.192	0.419	0.361	0.122
10	0.436	0.423	0.269	0.011	0.383
Mean	0.362	0.363	0.307	0.314	0.305
SD_r_	0.123	0.115	0.127	0.139	0.116
LOD: Mean + 3.3 SD_r_	0.768	0.742	0.725	0.772	0.686
Overall LOD = 0.738 mg/kg					
LOQest: Mean + 10 SD_r_	1.592	1.512	1.575	1.702	1.460
Overall LOQest = 1.568 mg/kg					

**Table 6.** GlutenTox ELISA Rapid G12—LOQ_est_ validation

Spiked matrixes at LOQ_est_: Concentration (mg/kg gluten)					
Replicate	Soy flour	Corn bread	Rolled oats	Seasoning mix	Evaporated milk
1	1.434	1.475	2.207	1.396	1.011
2	1.456	1.541	1.541	1.545	1.102
3	1.493	2.009	2.001	1.707	1.140
4	1.697	1.668	2.089	1.249	1.179
5	1.438	1.781	1.893	1.348	1.179
6	1.181	2.014	2.226	1.951	1.058
7	1.420	1.602	1.908	1.747	1.089
8	1.574	1.517	1.810	1.712	1.110
9	1.285	1.640	1.614	1.519	1.071
10	1.848	1.682	1.898	1.541	1.007
Mean	1.483	1.693	1.919	1.571	1.095
SD_r_	0.191	0.190	0.226	0.211	0.061
RSD_r_%	12.857	11.214	11.804	13.450	5.554
Recovery%	93	112	122	92	75
Overall LOQest = 1.552 mg/kg

- *Validation of the estimated LOQ (LOQ_est_).—*Each matrix was spiked with wheat flour at the LOQ_est_ (1.59 mg/kg soy flour, 1.51 mg/kg corn bread, 1.57 mg/kg rolled oats, 1.70 mg/kg seasoning mix, and 1.46 mg/kg evaporated milk), and 10 test portions were tested to demonstrate acceptable precision, RSDr of less than 20%.

Validation of the LOQ_est_ of each matrix produced average values of 1.48 mg/kg (12.86% RSD) for soy flour, 1.69 mg/kg (11.2% RSD) for corn bread, 1.92 mg/kg (11.8% RSD) for rolled oats, 1.57 mg/kg (13.4% RSD) for seasoning mix, and 1.09 mg/kg (5.55% RSD) for evaporated milk by GlutenTox ELISA Rapid G12, with a mean LOQ of 1.552 mg/kg across all matrixes tested.

### LOD and LOQ Determinations of Incurred Matrix (Processed Food)

Gluten-free bread matrix spiked with wheat, barley, and rye flours, separately, was baked and prepared by the independent laboratory.

LOD and LOQ were also estimated in 30 blank test portions of the gluten-free incurred bread matrix provided by the independent laboratory to perform the incurred matrix study. The LOD is expressed as the mean value of the negative sample determination (blank result) plus 3.3 SDs.

LOD was determined to be 0.193 mg/kg (10 blank test portions for the incurred bread matrix study with wheat flour), 0.333 mg/kg (10 blank test portions for the incurred bread matrix study with barley flour), and 0.215 mg/kg (10 blank test portions for the incurred bread matrix study with rye flour) by GlutenTox ELISA Rapid G12, with a mean LOD of 0.247 mg/kg across all test portions tested.

The LOQ is expressed as the mean value of the negative sample determination (blank result) plus 10 SDs.

LOQ was determined to be 0.414 mg/kg (incurred bread matrix study with wheat flour), 0.845 mg/kg (incurred bread matrix study with barley flour), and 0.493 mg/kg (incurred bread matrix study with rye flour) by GlutenTox ELISA Rapid G12, with a mean LOQ of 0.584 mg/kg across all test portions tested.

A summary of LOD and LOQ results is presented in Table 7.

**Table 7.** GlutenTox ELISA Rapid G12—LOD-LOQ_est_ study

Blank baked bread matrixes (incurred): Concentration (mg/kg gluten)			
Replicate	Wheat flour study	Barley flour study	Rye flour study
1	0.063	0.189	0.091
2	0.073	0.006	0.069
3	0.146	0.051	0.116
4	0.022	0.144	0.003
5	0.105	0.055	0.112
6	0.073	0.153	0.005
7	0.105	0.013	0.096
8	0.078	0.010	0.088
9	0.104	0.176	0.107
10	0.073	0.004	0.096
Mean	0.084	0.080	0.078
SD_r_	0.033	0.077	0.041
LOD: Mean + 3.3 SD_r_	0.193	0.333	0.215
Overall LOD = 0.247 mg/kg			
LOQest: Mean + 10 SD_r_	0.414	0.845	0.493
Overall LOQest = 0.584 mg/kg			

### Selectivity Study

The selectivity study was performed to demonstrate that the GlutenTox ELISA Rapid G12 method does not produce positive results, cross-reactivity, when tested on common food ingredients that do not contain any gluten, and at the same time demonstrate this method’s ability to detect target compounds (gluten from wheat, barley, and rye) without interference from the matrix when tested in the presence of gluten. The list of compounds recommended by *Guidelines for Validation of Quantitative Gluten Methods, with Specific Examples for ELISA Assays* was prescreened using the AOAC OMA **2012.01** method (5) to detect natural contamination prior to the study.

Each compound was purchased from a local or online store and was tested as it normally would be consumed (raw or cooked), based on full-strength extracts. Guar gum and xanthan gum were diluted in rice flour due to the viscous nature of the compounds.

Individual test portions of each compound without contamination (blank test portions) were prepared. Using a high concentration stock spiking material in rice flour, individual test portions of each compound with 20 mg/kg gluten from wheat flour were also prepared.

Blind-coded and randomized test portions (spiked and blank test portions) were tested once using the GlutenTox ELISA Rapid G12 test kit according to the method instructions. Any unspiked compounds that tested positive were required to be retested by testing six test portions of the compound with the GlutenTox ELISA Rapid G12 test kit.

All matrixes prescreened for AOAC OMA **2012.01** (5) produced a value of <5 mg/kg gluten (<LOQ) with the exception of oat flour (5.6 mg/kg gluten), romano bean flour (6.63 mg/kg gluten), fava bean flour (13.12 mg/kg gluten), and lima bean flour (>80 mg/kg gluten). The lima bean flour matrix was diluted 1:30 and retested using the AOAC OMA **2012.01** method (5), producing a value of 10.98 (329.4 mg/kg gluten); therefore, it was excluded from the interference study due to the high gluten content.

Each blank compound and gluten spiked compound were tested once according to the GlutenTox ELISA Rapid G12 test kit package insert. The results are shown in Table 8.

**Table 8.** GlutenTox ELISA Rapid G12—Selectivity study

Compounds	GlutenTox ELISA Rapid G12		
	Unspiked	20 ppm	
	Result (mg/kg gluten)	Result (mg/kg gluten)	Correctness
Almond flour	Below LOQ	17.69	−12%
Amaranth flour	Below LOQ	18.27	−11%
Arrowroot	Below LOQ	18.86	−6%
Black bean flour	Below LOQ	19.89	−1%
Brown rice flour	Below LOQ	19,82	−1%
Buckwheat flour	Below LOQ	21.06	5%
Chestnut flour	Below LOQ	21.09	5%
Coconut flour	Below LOQ	23.24	16%
Ground coffee	Below LOQ	19.76	−1%
Corn starch/meal	Below LOQ	20.54	3%
Dried fruits	Below LOQ	18.91	−5%
Egg powder	Below LOQ	20.37	2%
Fava bean flour	9.82	24.06	19%
Fava beans, ground^a^	Below LOQ	18.35	−8%
Flax seed flour	Below LOQ	22.09	10%
Green pea flour	Below LOQ	19.69	−2%
Guar gum (1:10)	Below LOQ	21.85	8%
Hazelnut flour	Below LOQ	19.18	−5%
Lentil flour	Below LOQ	19.09	−5%
Lima bean flour	297	–	–
Lima beans, ground^a^	Below LOQ	16.82	−16%
Milk powder	Below LOQ	20.44	1%
Milk (whole, liquid)	Below LOQ	14.82	−26%
Millet flour	Below LOQ	20.35	3%
Oat flour	2.79	17.38	−13%
Oats, rolled^a^	Below LOQ	20.85	4%
Parsley flakes	Below LOQ	22.14	10%
Pork sausage	Below LOQ	19.76	−3%
Potato starch	Below LOQ	23.71	19%
Quinoa flour	Below LOQ	19.18	−4%
Romano bean flour	6.06	23.68	18%
Romano beans, ground^a^	Below LOQ	18.53	−7%
Sorghum flour	Below LOQ	19.72	−1%
Soy flour	Below LOQ	22.76	13%
Sweet rice flour	Below LOQ	19.85	−1%
Tapioca flour	Below LOQ	19.11	−4%
Ground tea	Below LOQ	15.39	−23%
White bean flour	Below LOQ	15.68	−22%
White rice flour	Below LOQ	18.64	−7%
Xanthan gum (1:20)	Below LOQ	18.47	−9%
Yellow pea flour	Below LOQ	24.17	19%

a Indicates commodities ground into meal from bean/oat material and retested.

All matrixes tested using the GlutenTox ELISA Rapid G12 test kit gave results <0.6 mg/kg gluten (<LOQ) for the cross-reactivity study except for the same matrixes that tested positive in the prescreening evaluation with the AOAC OMA **2012.01** (5): oat flour (2.79 mg/kg gluten), romano bean flour (6.06 mg/kg gluten), fava bean flour (9.82 mg/kg gluten), and lima bean flour (297 mg/kg gluten). These matrixes were not retested with the GlutenTox ELISA Rapid G12 test kit since they had already produced positive results in the prescreening evaluation with the AOAC OMA **2012.01** (5). However, further analysis was carried out using the same matrixes in bean format (and rolled oats) and grinding them in the laboratory before performing the tests to minimize the risk of gluten contamination. Now the ground matrixes tested produced results <0.6 mg/kg gluten (<LOQ) for the cross-reactivity study (Table 8). For the 20 mg/kg spike level, all matrixes tested using the GlutenTox ELISA Rapid G12 test kit gave positive results in the interference study, ranging from 14.82 mg/kg gluten (milk: whole, liquid) to 24.17 mg/kg gluten (yellow pea flour).

### Additional Wheat Flour Cultivars

A total of six wheat flour varieties were obtained and were prepared according to the GlutenTox ELISA Rapid G12 test kit package insert, blind-coded, and tested once. For this purpose, six individual test portions of rice flour spiked with 20 mg/kg gluten from each variety of wheat flour were prepared. As the conversion factors of these varieties of wheat to convert percent protein to percent gluten were not available, we were recommended to use the conversion factor of 0.80 (usual factor of wheat flour “*Triticum aestivum or compactum*”) for the spiking calculations.

The results are shown in Table 9.

**Table 9.** GlutenTox ELISA Rapid G12—Selectivity study: Rice flour spiked at 20 mg/kg gluten from other sources of gluten

Compounds	GlutenTox ELISA Rapid G12		
	Unspiked	20 ppm	
	Result (mg/kg gluten)	Result (mg/kg gluten)	Correctness
Einkorn wheat flour (*Triticum monococcum*)	–	7.22	−64%
Khorasan wheat flour (*Triticum turgidum*)	–	18.29	−9%
Spelt wheat flour (*Triticum spelta*)	–	23.84	18%
Triticale flour (xTriticosecale)	–	8.39	−59%
Durum wheat flour (*Triticum durum*)	–	23.81	19%
Emmer wheat flour (*Triticum dicoccon*)	–	22.72	4%

**Table 10.** GlutenTox ELISA Rapid G12—Robustness study

Treatment combination	Reagent temperature	Extraction solution vortex time	ELISA HRP incubation time	Target contamination level, mg/kg	Mean concentration obtained, mg/kg	S_r_
1	4–8°C	5 s	20 min	0	<LOQ	
				10	8.835	0.866
2	4–8°C	5 s	40 min	0	<LOQ	
				10	8.581	0.909
3	4–8°C	60 s	20 min	0	<LOQ	
				10	8.412	0.854
4	4–8°C	60 s	40 min	0	<LOQ	
				10	8.688	0.503
5	30–40°C	5 s	20 min	0	<LOQ	
				10	7.692	1.066
6	30–40°C	5 s	40 min	0	<LOQ	
				10	8.171	0.800
7	30–40°C	60 s	20 min	0	<LOQ	
				10	7.171	0.456
8	30–40°C	60 s	40 min	0	<LOQ	
				10	7.511	0.719
9	15–25°C	30 s	30 min	0	<LOQ	
	Room temperature			10	7.632	0.848

**Table 11.** GlutenTox ELISA Rapid G12—Intermediate precision study (design 2b) in the incurred matrix from candidate

Lot 1									Lot 2								Lot 3							
	Day 1 Operator 1				Day 2 Operator 2				Day 1 Operator 1				Day 2 Operator 2				Day 1 Operator 1				Day 2 Operator 2			
	Ext/TP1		Ext/TP2		Ext/TP1		Ext/TP2		Ext/TP1		Ext/TP2		Ext/TP1		Ext/TP2		Ext/TP1		Ext/TP2		Ext/TP1		Ext/TP2	
	mg/kg	Recovery	mg/kg	Recovery	mg/kg	Recovery	mg/kg	Recovery	mg/kg	Recovery	mg/kg	Recovery	mg/kg	Recovery	mg/kg	Recovery	mg/kg	Recovery	mg/kg	Recovery	mg/kg	Recovery	mg/kg	Recovery
E1	**11.57**	**58%**	**12.12**	**61%**	**13.71**	**69%**	**12.79**	**64%**	**14.11**	**71%**	**12.97**	**65%**	**12.36**	**62**%	**12.46**	**62**%	**13.60**	**68**%	**11.06**	**55**%	**13.81**	**69**%	**13.76**	**69**%
	**10.67**	**53%**	**12.46**	**62%**	**13.02**	**65%**	**14.37**	**72%**	**13.48**	**67%**	**12.36**	**62%**	**12.35**	**62**%	**13.97**	**70**%	**12.57**	**63**%	**12.44**	**62**%	**14.09**	**70**%	**13.01**	**65**%
E2	**10.79**	**54%**	**10.93**	**55%**	**12.71**	**64%**	**11.77**	**59%**	**15.01**	**75%**	**13.16**	**66%**	**12.45**	**62**%	**10.35**	**52**%	**12.73**	**64**%	**9.59**	**48**%	**13.90**	**70**%	**12.59**	**63**%
	**9.75**	**49%**	**11.76**	**59%**	**12.78**	**64%**	**13.34**	**67%**	**14.44**	**72%**	**12.79**	**64%**	**12.18**	**61**%	**12.36**	**62**%	**11.54**	**58**%	**11.05**	**55**%	**14.03**	**70**%	**13.61**	**68**%

**Table 12.** GlutenTox ELISA Rapid G12—Validation statistics of the intermediate precision study (design 2b) in the incurred matrix from candidate: ANOVA table

Name	DF	SS	MS	VC	Total, %	SD	CV, %
Total	19.95045			1.694917	100	1.30189	10.33382
Lot	2	5.003117	2.501558	0	0	0	0
Lot: Calib	9	43.46895	4.829883	0.96975	57.2152	0.984759	7.81658
Lot: Calib: TP	12	11.4106	0.950883	0.225717	13.31727	0.475096	3.771106
Error	24	11.9868	0.49945	0.49945	29.46753	0.706718	5.609613
Mean: 12.59833 (*N* = 48)							

**Table 13.** GlutenTox ELISA Rapid G12—Validation statistics of the intermediate precision study (design 2b) in the incurred matrix from candidate: Standard deviation without replication (one test portion per sample and one ELISA well per test portion)

*n*	48
Mean	12.60 mg/kg
s(i)	1.30 mg/kg
RSD(i)	10.33%
s(r)	0.852 mg/kg
RSD(r)	6.76%

**Table 14.** GlutenTox ELISA Rapid G12—Validation statistics of the Intermediate precision study (design 2b) in the incurred matrix from candidate: Standard deviation with replication (one test portion per sample and two ELISA wells per test portion)

Mean	12.60 mg/kg
s(i)	0.779 mg/kg
RSD(i)	6.19%
s(r)	0.690 mg/kg
RSD(r)	5.47%

**Table 15.** GlutenTox ELISA Rapid G12—Stability accelerated and real-time studies

Stability study	Matrix	Storage temperature	Target contamination level, mg/kg	Concentration obtained, mg/kg Time points			Mean concentration obtained, mg/kg	Recovery	S_r_	RSD_r_
				8 days	32 days	40 days				
Accelerated	Rice flour	25 ± 2°C	0	<LOQ	<LOQ	<LOQ	<LOQ			
			10	7.18	7.84	8.02	7.68	77%	0.44	6%
			20	16.30	15.69	14.40	15.63	78%	0.70	4%

				3 months	9 months	15 months				

Real time	Rice flour		0	<LOQ	<LOQ	<LOQ	<LOQ			
			10	8.33	7.20	8.13	7.89	79%	0.60	8%
			20	15.26	17.55	16.31	16.37	82%	1.15	7%

For the 20 mg/kg spike level, all other sources of gluten in rice flour tested using the GlutenTox ELISA Rapid G12 test kit were detected and gave positive results, ranging from 7.22 mg/kg gluten (einkorn wheat flour (*Triticum monococcum*) to 23.84 mg/kg gluten (spelt wheat flour (*Triticum spelta*).

### Robustness Study

The robustness study was performed to evaluate the ability of the GlutenTox ELISA Rapid G12 method to remain unaffected by small variations in procedural parameters that might be expected to occur when the method is performed by an end user. Three parameters important to the end user were chosen to be varied. The effects of perturbations in reagent temperature (4–8ºC or 30–40ºC, normal = room temperature (15ºC–25ºC)), extraction solution vortex time (vortexing time; 5 or 60 s, normal = 30 s), and A1-HRP conjugate incubation time (20 or 40 min, normal = 30 min), were examined for rice flour test portions using the gluten-containing reference material (wheat flour). A factorial design was used to test the ruggedness parameters. Spikes were tested at two different spike levels of gluten (0 and 10 mg/kg).

For each spike level, 10 blind-coded replicate test portions were analyzed by the GlutenTox ELISA Rapid G12 method, but varying the parameters as indicated for each treatment combination.

Variations in the reagent temperature before starting the assay in combination with variations in the vortexing time of the extraction mixture before a further extraction incubation process and variations in the antibody conjugate incubation time, had little effect on results when compared to the optimal conditions (treatment combination 9).

Robustness results are presented in Table 10.

### Intermediate Precision Study

Intermediate precision of GlutenTox ELISA Rapid G12 test method was examined using the design 2b (Figure 27) and the results are presented in Table 11. The validation statistics are shown in Tables 12, 13 and 14.

Design 2b (Figure 27) was used to estimate *(1)* intermediate precision (which includes repeatability, test kit lot variance (with 2 degrees of freedom), day/operator confounded variance, and ELISA variance), *(2)* repeatability (which includes test portion variance and ELISA variance), *(3)* ELISA variance, and *(4)* lot-to-lot product consistency.

Incurred test material (gluten-free bread mix spiked with wheat flour) at 20 mg/kg spike level of gluten (middle level) was tested using three GlutenTox ELISA Rapid G12 test kit lots. Two operators conducted analysis on two days for each test kit lot. Each day, the assigned operator conducted analysis of two individually extracted test portions of test material, with the ELISA measurements performed in duplicate for each.

Single Lab Validation set from GlutenTox ELISA Rapid G12 test method was analyzed for validation statistics. The method validation design used 3 kit lots, 2 analysts over 2 days, with 2 test portions per sample and 2 ELISA per test portion. This is described as AOAC GFA Design design 2b, with modification that the end point determination (the ELISA measurement) was performed in duplicate (per GlutenTox instructions), that is, the design was run twice. Since 12 calibrations were performed, it was decided to analyze Analyst/Day/Calibration as a single confounded factor as a stand-in variable for these intermediate factors. This confounded “Calibration” factor was deemed to be nested within “Lot”, so a fully nested analysis model was used here. This was a single test material at an approximate detection level of 12 mg/kg gluten.

In this study, for this matrix, the nested ANOVA table (Table 12) shows the greatest contribution to overall variance is the confounded factor – (Day/Analyst/Calibration) at 57.2% of the variance. The second highest was ELISA (error) at 29.5% of total variance. Interestingly, kit Lot was very small (0%) contributor to overall variance. The variance of the final method can be estimated by mathematically dividing the variances of the components by the number of replicates of the study. In this case, the overall RSD for the method is 10.33% (sd(i) = 1.30) (Tables 12 and 13) which is mathematically reduced to 6.19% (by over 4%) (sd(i) = 0,779) (Table 14) when the variance of the ELISA component (0.49945) is divided by the number of replicates (two ELISA wells per test portion) tested (obtaining a value of 0.249725 for the variance of the ELISA component).

### Stability Study

Stability of GlutenTox ELISA Rapid G12 test kit was examined in rice flour matrix through accelerated studies based on the Arrhenius model (6), having to support the shelf life of 12 months. In this regard, one lot of the GlutenTox ELISA Rapid G12 test kit contents were stored at 25ºC (2–8ºC is the normal storage temperature of the GlutenTox ELISA Rapid G12 test kit contents) and the accelerated stability study was performed testing the kit at specified time points [8 days (equivalent to 3 months), 32 days (equivalent to 9 months), and 40 days (equivalent to 15 months)]. Rice flour matrix spiked with wheat flour at three different spiked levels of gluten (0 mg/kg, 10 mg/kg and 20 mg/kg) were prepared using the same preparation techniques as used in the matrix study. For each spiked level of gluten and at each stability time point, 5 blind coded replicate test portions were analyzed using the GlutenTox ELISA Rapid G12 test method.

As accelerated stability provides only a rough estimate of shelf-life, real time data supporting the entire shelf life of the kit under normal storage conditions (2–8ºC) was also performed in the same matrix (rice flour) and spiked levels of gluten (0 mg/kg, 10 mg/kg and 20 mg/kg) and following the same procedure as the accelerated study but at different specified time points (3 months, 9 months, and 15 months).

Accelerated and real time stability results are presented in Table 15.

#### Accelerated stability time point: 8, 32 and 40 days

The evaluation of sample extracts for recovery tested at on kit lots aged by acceleration for 8, 32 and 40 days produced average values of <LOQ recovery at 0 mg/kg spike level of gluten, 77% recovery at 10 mg/kg spike level and 78% recovery at 20 mg/kg spike level.

The evaluation of the same sample extracts for repeatability produced average values of 6% RSD at 10 mg/kg and 4% RSD at 20 mg/kg spike level of gluten.

#### Real time stability time point: 3, 9 and 15 months

The evaluation of sample extracts for recovery tested at on kit lots aged by real time for 3, 9 and 15 months produced average values of <LOQ recovery at 0 mg/kg spike level of gluten, 79% recovery at 10 mg/kg spike level and 82% recovery at 20 mg/kg spike level.

The evaluation of the same sample extracts for repeatability produced average values of 8% RSD at 10 mg/kg and 7% RSD at 20 mg/kg spike level of gluten.

The real time stability data (3 months, 9 months, and 15 months at 2–8ºC) were consistent with the results previously obtained in the accelerated stability studies.

### Independent Laboratory Studies

The study was conducted following the procedures outlined in the AOAC Research Institute *Performance Tested Methods*^SM^ protocol: *Independent Validation Protocol for the GlutenTox ELISA G12 Kit*, version 3.2, April 21, 2022. The study evaluated the GlutenTox ELISA Rapid G12 test kit in a non-heat-processed food matrix study and an incurred or heat processed matrix study. The food matrix study evaluated corn bread and seasoning mix contaminated with gluten from wheat flour at four levels: 0, 5, 10, and 20 mg/kg. Ten individually extracted test portions were evaluated for each matrix and contamination level. Samples were tested by two analysts on two instruments for each contamination level (blind-coded) according to the kit instructions. The mean concentration at each level was used to calculate the bias, recovery, repeatability precision determination (RSD_r_), and LOD and LOQ.

For the incurred matrix testing, 10 individually extracted test portions of baked gluten-free bread were tested in the same fashion as the food matrix for each level and each contamination source. The baked gluten-free bread was incurred with wheat, barley, or rye flour at three levels: 0, 20, and 30 mg/kg. Samples were tested by two analysts on two instruments for each contamination level (blind-coded) according to the kit instructions. Following analysis, results were decoded and used to calculate the mean concentration at each level, which, in turn was used to calculate the bias, recovery, repeatability precision determination (RSD_r_), and LOD and LOQ.

**Figure 28.**
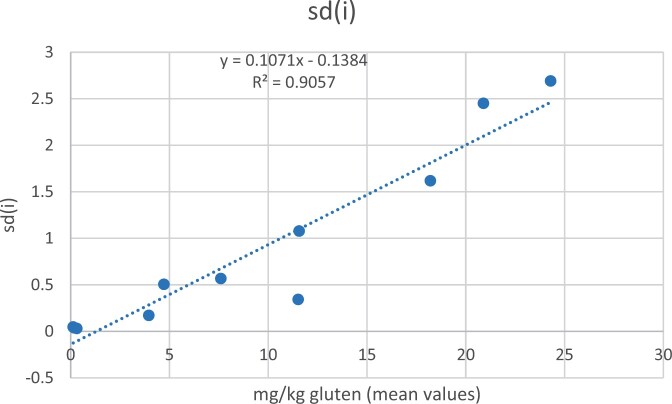
Intermediate precision standard deviations at five different concentrations for three different matrixes from the independent laboratory.

The whole number of matrixes and test portions were used to calculate sd*_i_* (Table 16). From these, all matrixes contaminated with wheat flour at 0, 5, 10, 20, and 30 mg/kg of gluten were used to calculate the LOD according to the linear regression model (Figure 28) and the following formula: $\mathrm{LOD}=({\bar{x}}_{\left(0\right)}+3.3\times S_{r\left(0\right)})/(1-1.65\times \mathrm{slope})$. A pooled standard deviation at 0 ppm level of 0.041, with a mean at zero of 0.20 mg/kg, and then a slope of 10%. These data were put in a calculator, and an LOD estimate of 0.4 mg/kg was obtained.

**Table 16.** Intermediate precision standard deviation, Sd(i), calculation for each matrix/grain/spiking level combination from the independent laboratory

Matrix	Grain	Level	n	Mean	sd(i)
Bread	Wheat	0	20	0.127	0.047603
Seasoning	Wheat	0	12	0.179167	0.037528
Toast	Wheat	0	12	0.310833	0.031314
Seasoning	Wheat	5	12	3.9525	0.172342
Toast	Wheat	5	12	4.719167	0.505676
Toast	Wheat	10	12	7.6025	0.567188
Seasoning	Wheat	10	12	11.515	0.343565
Bread	Wheat	20	20	11.568	1.078596
Bread	Wheat	30	20	18.2075	1.618019
Toast	Wheat	20	12	20.89583	2.451214
Seasoning	Wheat	20	12	24.29667	2.691233

An LOQ of 1.2 mg/kg was obtained according to the formula: LOQ = 3 × LOD

Tables 2 and 4 provide a summary of mean, bias, recovery, and repeatability precision determination obtained for each food matrix and incurred matrix, respectively, for analysts 1 and 2.

## Discussion

The GlutenTox ELISA Rapid G12 method did not show cross-reactivity to any of the compounds included in the list of *Guidelines for Validation of Quantitative Gluten Methods, with Specific Examples for ELISA Assays* or those added to the AOAC Research Institute *Performance Tested Methods*^SM^ program: *PTM Validation of the GlutenTox ELISA Rapid G12 Kit in Select Foods*, version 10, September 27, 2021, used in the production of gluten-free products. Four compounds that had tested positive in the prescreening evaluation with the AOAC OMA **2012.01** (5) (oat flour, romano bean flour, fava bean flour, and lima bean flour) also tested >LOQ with the GlutenTox ELISA Rapid G12 test kit and were not retested. To determine if the positivity of these matrixes was due to gluten contamination during the manufacturing process or to a cross-reaction, further analysis was carried out using the same matrixes in bean format (and rolled oats) and grinding them in the laboratory before performing the tests to minimize the risk of a gluten contamination. Definitively, the GlutenTox ELISA Rapid test kit did not show cross-reactivity with these matrixes. Therefore, it can be confirmed that the previous positive results were due to gluten contamination. The GlutenTox ELISA Rapid G12 assay also did not show any interference, when tested with the required compounds for testing in the presence of gluten. No unexpected results were obtained (the lima bean matrix included in the interference study was that in bean format and subsequently ground).

The GlutenTox ELISA Rapid G12 test kit performed as expected when six additional wheat flour varieties were tested in rice flour, and positive results were obtained in all wheat cultivars analyzed. However, with the einkorn wheat flour (*Triticum monococcum*) variety, a recovery result lower than expected was obtained. Further studies are needed to determine if this is due to a lower gluten: protein ratio.

The lot-to-lot data (from the intermediate precision study), the accelerated stability data (8 days, 32 days, and 40 days at 25°C), and the real-time stability data (3 months, 9 months, and 15 months at 2–8°C) showed evidence that the GlutenTox ELISA Rapid G12 method is stable and can be consistently manufactured with reproducible quality.

Robustness data indicated that the GlutenTox ELISA Rapid G12 assay remained unaffected by minor variations in procedural parameters.

The GlutenTox ELISA Rapid G12 test kit performed as expected in the selected food matrixes (gluten-free soy flour, corn bread, seasoning mix, rolled oats, and evaporated milk), spike levels of gluten with wheat flour, and in both Hygiena (method developer) and the independent laboratory (only the corn bread and seasoning mix matrixes were tested), obtaining comparable results.

In all matrixes tested at different spike levels with barley and rye flours, the GlutenTox ELISA Rapid G12 assay performed as expected (meeting performance claims for recovery and repeatability, mainly with barley flour) or showing slight (<28%) to moderate (46% or 85%) overestimation depending on the matrix, source of gluten contaminant, and gluten concentration.

Results obtained in the method developer incurred matrix study with wheat, barley, and rye flours indicate that the assay performed as expected (meeting performance claims for recovery and repeatability, mainly with wheat and rye flours) or showing slight (25% or 37%) to moderate (49%) overestimation depending on the source of gluten contaminant and gluten concentration. These data are comparable to those obtained in the incurred sample study of the independent laboratory where the GlutenTox ELISA Rapid G12 method performed as expected with wheat and rye flours and showed a slight (11%) to moderate (77%) overestimation with barley at 20 mg/kg and 30 mg/kg spike levels of gluten, respectively.

Nevertheless, this occasional overestimation of gluten from barley or rye is a less important factor in gluten analysis for the people with celiac disease, since possible problems from false negatives or underestimations could be much worse. No false negative results were observed in the entire validation study.

The GlutenTox ELISA Rapid G12 assay performed as expected in the calibration study in all dilutions. To minimize the trend of a nonrandom pattern found in the higher analyte concentrations of each dilution, a suitable dilution should be made according to the expected amount of gluten in the sample.

The intermediate precision study demonstrated that the design 2b and the contribution of the analyst/day/calibration as a single confounded factor to the variance were appropriate when the GlutenTox ELISA Rapid G12 assay was tested with the incurred bread matrix.

In this study, the overall RSD for the method was in accordance with the acceptance criteria, and even was mathematically reduced by over 4% when the variance of the ELISA component was divided by the number of replicates tested (two ELISA wells per test portion).

The overall validation of the estimated LOQ (LOQ_est_) of the GlutenTox ELISA Rapid G12 test kit by the method developer in the selected matrixes performed as expected, showing an excellent correlation with the overall LOD-LOQ_est_ (according to the standard deviation of blank samples). These results are in line and are consistent with the LOD and LOQ values obtained from the independent laboratory (calculated from the linear regression model) using three matrixes and four spike concentration levels of gluten from wheat flour (LOD = 0.4 mg/kg gluten and LOQ = 1.2 mg/kg gluten).

## Conclusions

The GlutenTox ELISA Rapid G12 test kit is a quick and easy-to-use method for the detection and quantification of gluten concentration in food and incurred matrixes from wheat, barley, and rye flours.

The method is specific and reliable and provides sensitive and accurate test results, showing occasional slight to moderate overestimation depending on the matrix and gluten concentration from barley and rye flours, and it should be granted PTM certification.

The GlutenTox ELISA Rapid G12 test kit is a stable and cost-effective kit recommended for laboratories and industry. The instructions for use include information about the preparation of different dilutions depending on the expected gluten content of the sample.
